# Single‐Cell Spatial Transcriptomics Unveils Platelet‐Fueled Cycling Macrophages for Kidney Fibrosis

**DOI:** 10.1002/advs.202308505

**Published:** 2024-06-05

**Authors:** Jun Liu, Bo Zheng, Qingya Cui, Yu Zhu, Likai Chu, Zhi Geng, Yiming Mao, Lin Wan, Xu Cao, Qianwei Xiong, Fujia Guo, David C Yang, Ssu‐Wei Hsu, Ching‐Hsien Chen, Xiangming Yan

**Affiliations:** ^1^ Pediatric Institute of Soochow University Children's Hospital of Soochow University Soochow University Suzhou 215025 China; ^2^ State Key Laboratory of Reproductive Medicine and Offspring Health The Affiliated Suzhou Hospital of Nanjing Medical University Suzhou Municipal Hospital Gusu School of Nanjing Medical University Suzhou 215002 China; ^3^ National Clinical Research Center for Hematologic Diseases Jiangsu Institute of Hematology The First Affiliated Hospital of Soochow University Suzhou 215006 China; ^4^ Department of Thoracic Surgery Suzhou Kowloon Hospital Shanghai Jiao Tong University School of Medicine Suzhou 215028 China; ^5^ Department of Microbiology Immunology & Molecular Genetics University of California Los Angeles CA 90095 USA; ^6^ Department of Internal Medicine Division of Nephrology University of California Davis CA 95616 USA; ^7^ Department of Internal Medicine Division of Pulmonary and Critical Care Medicine University of California Davis Davis CA 95616 USA

**Keywords:** kidney fibrosis, macrophage proliferation, platelet, thrombospondin 1

## Abstract

With the increasing incidence of kidney diseases, there is an urgent need to develop therapeutic strategies to combat post‐injury fibrosis. Immune cells, including platelets, play a pivotal role in this repair process, primarily through their released cytokines. However, the specific role of platelets in kidney injury and subsequent repair remains underexplored. Here, the detrimental role of platelets in renal recovery following ischemia/reperfusion injury and its contribution to acute kidney injury  to chronic kidney disease transition is aimed to investigated. In this study, it is shown that depleting platelets accelerates injury resolution and significantly reduces fibrosis. Employing advanced single‐cell and spatial transcriptomic techniques, macrophages as the primary mediators modulated by platelet signals is identified. A novel subset of macrophages, termed “cycling M2”, which exhibit an M2 phenotype combined with enhanced proliferative activity is uncovered. This subset emerges in the injured kidney during the resolution phase and is modulated by platelet‐derived thrombospondin 1 (THBS1) signaling, acquiring profibrotic characteristics. Conversely, targeted inhibition of THBS1 markedly downregulates the cycling M2 macrophage, thereby mitigating fibrotic progression. Overall, this findings highlight the adverse role of platelet THBS1‐boosted cycling M2 macrophages in renal injury repair and suggest platelet THBS1 as a promising therapeutic target for alleviating inflammation and kidney fibrosis.

## Graphical abstract

This study explores the role of platelets in renal fibrosis following ischemia/reperfusion injury, particularly during the transition from acute kidney injury to chronic kidney disease. It is discovered that platelet depletion reduces fibrosis and identified a subset of proliferative M2 macrophages as key mediators influenced by platelet‐derived THBS1. Inhibiting THBS1 suppresses these macrophages, offering a promising therapeutic target for kidney fibrosis.

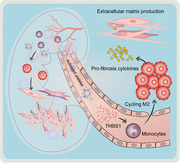

## 1 Introduction

Acute kidney injury (AKI) is a significant medical issue, primarily characterized by rapid renal dysfunction, often due to ischemia/reperfusion (I/R) injury.^[^ ^1^, ^2^ ^]^ Ischemia damages the tubular epithelial cells (TECs) initially with reperfusion further aggravating this damage, causing TECs to release damage‐associated molecular patterns that attract innate immune cells to the site of injury, and initiating an inflammatory cascade.^[^ ^3^, ^4^ ^]^ This damage and subsequent inflammation can trigger a sequence of events in the kidneys leading to progression toward chronic kidney disease (CKD) and ultimately fibrosis. The process is intricately modulated by various cellular and molecular pathways with one notable player being transforming growth factor‐beta 1 (TGF‐β1). This factor is released from renal TECs and plays a pivotal role in several key mechanisms such as autophagy and extracellular matrix deposition, contributing to the complex pathways involved in the transition of kidney injury to fibrosis.^[^ ^5^, ^6^ ^]^

The transition from AKI to CKD is mediated in a large part by aberrant tissue repair post‐ischemia/reperfusion injury (IRI). Prior studies have shown that severity of AKI, indicated by elevated markers such as serum creatinine, is associated with higher CKD risk.^[^ ^7^, ^8^ ^]^ The damage due to AKI and subsequent dysregulated repair processes can induce sustained inflammation, ultimately leading to CKD. A significant player in this inflammatory process are macrophages which have emerged as pivotal players in this process. During the initial stages of AKI, the environment is dominated by pro‐inflammatory M1 macrophages with a later shift toward pro‐repair M2 macrophages, with previous studies subdividing M2 into M2a, M2b, and M2c phenotypes.^[^ ^9^, ^10^ ^]^ During the progression of AKI to CKD, there is a shift in the populations of macrophages with M1 macrophages exacerbating renal injury, followed by anti‐inflammatory M2c macrophages fostering repair. However, dysregulated repair promotes profibrotic M2a macrophages, which significantly contribute to kidney fibrosis.^[^ ^10^, ^11^ ^]^ Recent research has highlighted several significant pathways in renal fibrosis, particularly the macrophage‐myofibroblast transition influenced by TGF‐β/Smad3 signaling.^[^ ^12^, ^13^ ^]^ Despite these advancements, a comprehensive understanding of macrophages' multifunctional roles in renal injury and repair remains incomplete.

One cell type that may play a significant role in inflammation and repair during AKI‐CKD is the platelet. Emerging research underscores the pivotal role of platelets in inflammation and immune responses, notably in vascular inflammatory conditions and AKI.^[^ ^14^, ^15^ ^]^ During AKI, elevated platelet activation contributes to enhanced activation and diminished regulation of inflammation.^[^ ^15^ ^]^ Although some prior studies have demonstrated no link between platelet levels and AKI,^[^ ^16^ ^]^ the role of platelets in AKI to CKD transition remains poorly understood. the platelet‐derived high‐mobility group box 1 This factor has been implicated in multiple inflammatory diseases, and its exogenous introduction in ischemia/reperfusion‐induced AKI models intensifies renal damage.^[^ ^17^ ^]^ Moreover, platelet‐derived CXCL4 is central in driving profibrotic macrophage activation and subsequent renal fibrosis.^[^ ^18^ ^]^

Furthermore, platelets have been shown to be able to influence macrophage polarization toward a pro‐inflammatory phenotype in the context of sepsis.^[^ ^19^ ^]^ Platelets have also been shown to play a similar pathological role in CKD through modulation of inflammation through secreted factors and interaction with immune cells including macrophages.^[^ ^20^ ^]^ This is mediated through secretion of both pro‐inflammatory factors such as IL‐1β, CD40 Ligand (CD40L), and CCL5 as well as pro‐fibrotic factors such as TGF‐β and PDGFs. One factor that has been of interest in AKI is the matricellular protein Thrombospondin‐1 (THBS1, also referred to as TSP‐1), released by activated platelets. THBS1 has been reported to play a crucial role in cellular interactions during tissue stress.^[^ ^21^ ^]^ Modulating its levels has been shown to be protective against sepsis‐induced AKI via the TGF‐β/Smad3 pathway.^[^ ^22^ ^]^ Furthermore, it has been identified as a mediator of pyroptosis in ischemia/reperfusion‐induced AKI.^[^ ^23^ ^]^ Prior findings have confirmed the function of THBS1 in promoting AKI through binding and activation of CD47 leading to recruitment of immune cells, promoting oxidative damage, and epithelial cell death.^[^ ^24^, ^25^, ^26^, ^27^ ^]^ These studies have demonstrated that targeting of THBS1/CD47 downregulates these activities and may be effective in attenuating damage associated with AKI. Taken together, these prior findings highlight the significant influence of platelets in AKI and CKD. However, the role that THBS1 plays in mediating the AKI‐CKD transition is still not completely understood.

In this study, we aimed to elucidate the role of platelets and their mechanistic effects on a newly identified macrophage subset during I/R and subsequent renal fibrosis. Using single‐cell and spatial transcriptomics analysis, we discovered that platelet‐derived THBS1 stimulates macrophage differentiation into a unique, highly proliferative M2‐like subtype. This distinctive M2‐like macrophage subset exacerbates kidney fibrosis by producing excessive ECM components. Collectively, our findings align with prior work to suggest that THBS1 holds promise as a potential therapeutic target for combating kidney fibrosis resulting from AKI.

## 2 Results

### 2.1 Platelets Link to AKI Severity and Play a Role in Ischemia/Reperfusion‐Induced Injury

**Figure 1:**
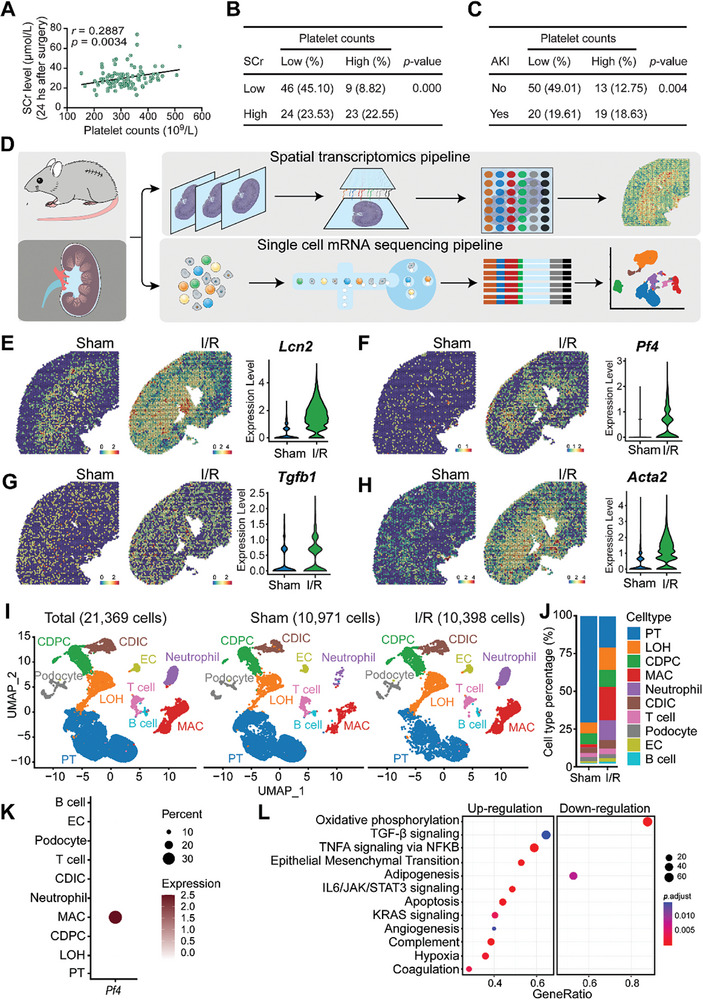
Platelets link to AKI severity and play a role in ischemia/reperfusion‐induced injury A total of 102 patients with diverse cardiovascular diseases who underwent the operation of extracorporeal circulation during cardiovascular surgery were recruited. All the patients were at risk for ischemia/reperfusion (I/R) AKI. A) The correlation of basal platelet counts and serum creatinine (SCr) level in the 102 patients were calculated; the 95% confidence interval (CI) was: 0.0988 to 0.4566. B) Correlation of patients with high‐ or low‐level of Scr and high‐ or low‐level of platelet counts in the cohort of 102 patients; a two‐tailed Fisher exact probability test was performed. C) Correlation of patients with or without AKI and high‐ or low‐level of platelet counts in the cohort of 102 patients; a two‐tailed Fisher exact probability test was performed. Wild‐type C57BL/6J mice were subjected to bilateral I/R injury surgery, or a sham operation, and were sacrificed 48 h after the surgery. Injured and normal sham kidneys were harvested for single‐cell mRNA‐sequencing and spatial transcriptomics. D) The single‐cell spatial transcriptomics experimental scheme on mouse kidney with or without I/R injury. E‐H) Spatial feature plots of *Lcn2*, *Pf4*, *Tgfb1*, and *Acta2* in ST spots and violin plots showing its relative expression level in sham or I/R‐treated mouse kidneys. I) UMAP projection of a total of 21 369 cells including 10 971 cells from the sham kidney, and 10 398 cells from the injured kidney; cell identity was annotated based on cell type‐specific markers. J) The barplot shows the percentage of each cell type out of the total cells in each group. K) Dotplot shows the expression pattern of platelet activation marker Pf4 in each cell type of the two groups. L) Dotplot shows the enriched GSEA hallmark terms in macrophages from I/R‐treated kidneys compared to sham kidneys based on the scRNA‐seq data. PT, proximal tubular; LOH, Loop of Henle; CDPC, collecting duct principal cell; MAC, macrophage; CDIC, collecting duct intercalated cell; EC, endothelial cell.

To elucidate the potential association between AKI and platelet counts, a cohort of 102 patients with diverse cardiovascular pathologies who underwent extracorporeal circulation procedures during cardiovascular surgery were analyzed. Each individual in this cohort was predisposed to ischemia/reperfusion (I/R) AKI. In our initial analysis, there was a significantly positive correlation between basal platelet counts and serum creatinine (SCr) levels, with a 95% confidence interval (CI) of 0.0988 to 0.4566 (**Figure** **1**A). This correlation suggests a potential mechanistic link between renal dysfunction, as indicated by elevated SCr levels, and platelet activity. Subsequent stratification based on SCr concentrations and platelet counts underscored this finding. There was a distinct positive association between heightened SCr levels and elevated platelet counts within the patient cohort (Figure 1B). Moreover, when patients were categorized based on the presence or absence of AKI and corresponding platelet counts, there was a marked association between the incidence of AKI and elevated serum creatinine levels (Figure 1C).

Driven by the observed association between platelets and AKI, we sought to comprehensively explore the molecular alterations in kidneys subjected to I/R using both spatial and single‐cell transcriptomics (Figure 1D). Through the analysis of spatial transcriptomics in injured kidneys (Figure S1A, Supporting Information), we noted elevated expression of the inflammatory marker *Lcn2*, the platelet activation marker *Pf4*, and the profibrotic markers *Tgfb1* and *Acta2* in I/R‐treated mouse kidneys among the molecules associated with tissue injury and repair (Figure 1E–H; Figure S1B–I, Supporting Information). Subsequently, single‐cell sequencing (scRNA‐seq) data encompassed a total of 21369 cells, which included 10971 cells from the sham kidney and 10398 cells from the I/R‐treated kidney. Cell identities were annotated based on specific markers (Figure 1I). A marked decrease in proximal tubular cells and a corresponding increase in macrophages were observed following AKI induction (Figure 1J). Detailed expression analysis of the platelet activation marker, platelet factor 4 (*Pf4*), across different cell types in both the AKI and sham groups revealed its exclusive expression in macrophages (Figure 1K; Figure S1J, Supporting Information). Pathway analysis identified a pronounced upregulation of several profibrotic pathways in the AKI group, most notably TGF‐β, IL6/JAK/STAT3, epithelial‐mesenchymal transformation (EMT), and the pro‐proliferative KRAS signaling pathways, three days after the injury (Figure 1L). Altogether, our findings indicate a strong correlation between elevated platelet counts and AKI, supported by spatial transcriptomics highlighting profibrotic pathways in IRI.

### 2.2 Depletion of Platelets Attenuates I/R‐Induced Kidney Damage and Fibrosis

**Figure 2:**
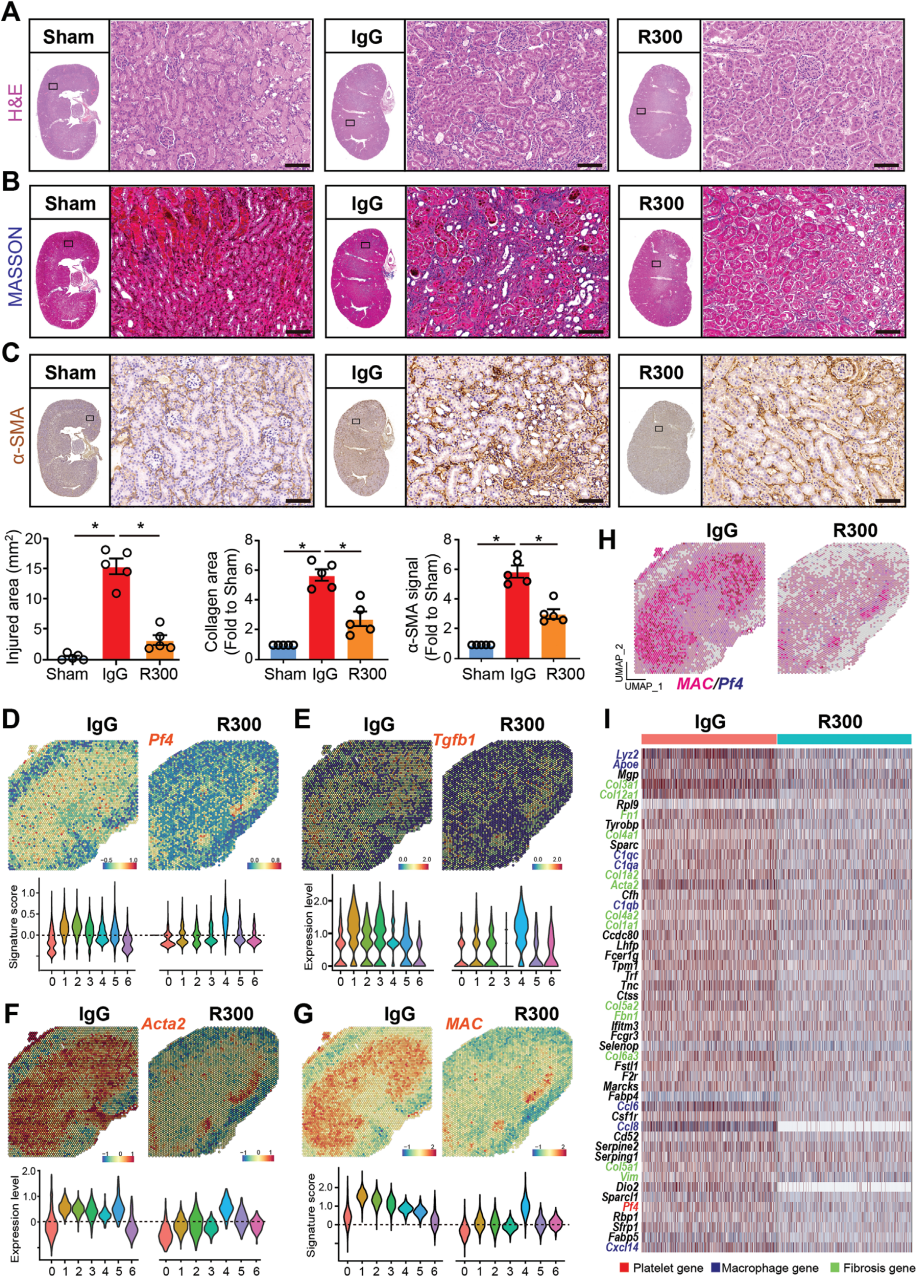
Depletion of platelets attenuates I/R‐induced kidney damage and fibrosis. Wild‐type C57BL/6J mice were subjected to bilateral I/R surgery on the kidney for 48 h followed by intravenous tail vein injection of 4 µg kg^−1^ mouse platelet depleting antibody R300 or the isotope IgG (for 4 days. Mice which received the sham operation were labeled as Sham (n = 5 mice per group). On the 7th day after the surgery, all the mice were sacrificed and kidney tissues were harvested for histopathology, single‐cell‐RNA‐sequencing, and spatial transcriptomics experiments. The paraffin‐embedded kidney sections were prepared and then stained to evaluate kidney injury and fibrosis progression. A–C) Representative images and quantification of H&E, MASSON staining (blue signal), and α‐SMA immunohistochemistry (IHC) signal in the kidney. The whole slide images of the serial sections of the stained kidney were also displayed, and differences between the indicated groups were analyzed using an unpaired two‐tailed Student's t‐test, and the statistical significance was labeled with an asterisk; Scale bar: 50 µm, *n* = 5 for each group. D–G) Spatial feature plots of *Pf4*, *Tgfb1*, *Acta2*, and macrophage signature (MAC) in spatial transcriptomics (ST) spots of the indicated groups and Violin plots showing its relative expression in each cell cluster. H) Feature plot showing the mRNA colocalization of *Pf4* and macrophage (MAC) signature genes, including *Adgre1, Itgax, Lyz2, Apoe, C1qb*, and *C1qa* in ST spots of the indicated two groups. I) The spatial expression matrix data of macrophage‐enriched clusters 1, 2, and 3 in the IgG‐treated kidney and the matched clusters 1 and 2 in the R300‐treated kidney were compared to define the differentially expressed genes (DEGs). Heatmap showing the expression of the top 50 genes upregulated in the IgG‐treated kidney. Platelet, macrophage, and fibrosis‐related genes are highlighted in red, blue, and green font respectively.

To elucidate the impact of platelets on kidney injury resolution, wild‐type C57BL/6J mice were subjected to bilateral I/R surgery on their kidneys for 48 h. Post‐surgery, these mice received an intravenous tail vein injection of either 4 µg kg^−1^ mouse platelet‐depleting antibody R300 (hereafter termed “R300”) or the isotype IgG (hereafter termed “IgG”) for 4 days. Sham‐operated mice served as controls (Figure S2A, Supporting Information). The efficiency of R300 in platelet depletion was evidenced by the decreased count of circulating platelets (Figure S2B, Supporting Information). By the 7th day post‐surgery, kidney tissues were collected for various analytical methods including histopathology, scRNA‐seq, and spatial transcriptomics. Histopathological analyses demonstrated marked structural damage in the kidneys of mice subjected to I/R surgery and treated with IgG. These changes were evident with increased interstitial fibroblasts, and an influx of immune cells (**Figure** **2**A). Moreover, Masson's trichrome staining of these kidneys exhibited pronounced interstitial collagen accumulation, indicative of fibrosis (Figure 2B). The heightened fibrosis was further corroborated by an increased α‐SMA immunohistochemistry (IHC) signal in the same group (Figure 2C). Strikingly, kidneys of I/R‐operated mice that received the R300 treatment presented with significantly attenuated histological damage, reduced fibrosis, and diminished α‐SMA signals, suggesting that platelet depletion ameliorates kidney injury and impedes subsequent fibrosis development.

Spatial transcriptomic analyses revealed notable downregulation of the genes *Pf4, Tgfb1, Acta2*, and the macrophage signature in the kidneys of I/R‐operated mice treated with R300 (Figure 2D–G; Figure S2C, Supporting Information). This downregulation was concomitant with the attenuation of profibrotic pathways such as TGF‐β, EMT, and pro‐proliferative KRAS signaling post‐R300 treatment (Figure S2D, Supporting Information). Intriguingly, while kidneys subjected to I/R displayed augmented mRNA colocalization of *Pf4* with macrophage signatures, this phenomenon was conspicuously absent in R300‐treated I/R‐operated kidneys (Figure 2H). Differential gene expression analysis of macrophage‐enriched clusters from IgG‐ and R300‐treated kidneys post I/R surgery demonstrated multiple genes upregulated in the IgG‐treated group compared to the R300‐treated group. This upregulation prominently featured genes associated with macrophages, fibrosis, and platelets (Figure 2I). In a subsequent exploration focusing on cluster 4 of the R300‐treated kidney, which interestingly showed the presence of platelets, we identified upregulation of macrophage‐, fibrosis‐, and platelet‐associated genes compared to other clusters (Figure S2E, Supporting Information). Collectively, these data underscore the importance of platelets in modulating AKI resolution.

### 2.3 Platelet‐Derived THBS1 Signaling Mediates Macrophage‐Fibroblast Communication in I/R‐Induced Kidney Fibrosis

**Figure 3:**
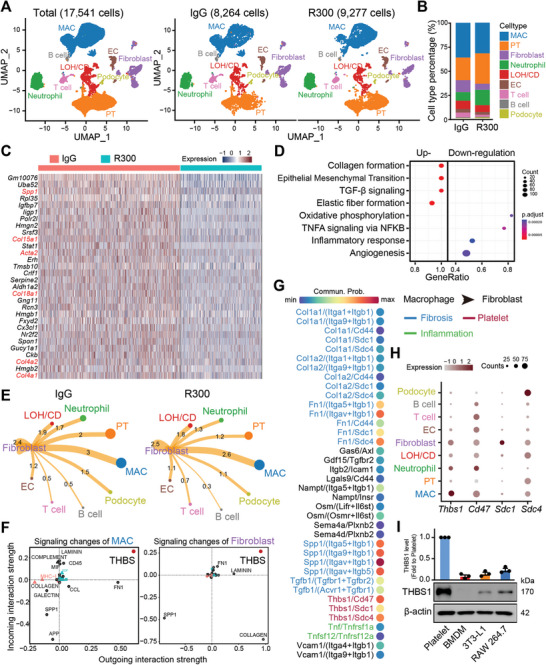
Platelet‐derived THBS1 signaling mediates macrophage‐fibroblast communication in I/R‐induced kidney fibrosis. Wild‐type C57BL/6J mice were subjected to surgery of bilateral I/R kidney injury for 48 h followed by intravenous injection of 4 µg kg^−1^ mouse platelet depleting antibody R300 or the isotope IgG for 4 days. On the 7th day after the surgery, mice were sacrificed, and kidney tissues were harvested for single‐cell‐RNA‐sequencing experiments. A) UMAP projection of a total of 17 541 cells including 8264 cells from the IgG‐treated injured kidney, and 9277 cells from the R300‐treated injured kidney; cell identity was annotated based on cell type‐specific markers. B) Bar plot of different cell type populations displayed as percentage of total cells. C) Heatmap showing the top 30 genes upregulated in fibroblast of the IgG‐treated injured kidney as compared to the R300‐treated kidneys. D) The dot plot shows the signaling pathways enriched in fibroblast of the IgG‐treated injured kidney as compared to the R300‐treated kidney based on the scRNA‐seq data in (C). E) The potential intercellular cell‐cell communications were predicted based on the interactive ligand‐receptor numbers by using the R package “Cellchat”. F) The possible outgoing and incoming signaling enriched in macrophage (left panel) and fibroblast (right panel) of the IgG‐treated injured kidney as compared to the R300‐treated kidney. G) The total pairs of signaling (ligand/receptor) potentially arising from macrophage outgoing to the fibroblast. H) The dot plot shows the expression pattern of *Thbs1* and its receptor genes in all identified cell types. I) The relative protein expression of THBS1 in platelet, bone marrow‐derived macrophage (BMDM), fibroblast cell line 3T3‐L1, and mouse monocyte/macrophage cell line RAW 264.7. Quantification of THBS1 is normalized to β‐actin with respect to the platelet group (n = 3; mean ± s.e.m.).

To investigate the cellular and molecular dynamics in response to platelet depletion during I/R‐induced kidney injury, both IgG‐ and R300‐treated kidneys 7 days post I/R surgery as described above were harvested and subjected to scRNA‐seq analysis. A UMAP projection of 17 541 cells illustrated distinct cellular landscapes: 8264 cells from the IgG‐treated injured kidney and 9277 cells from the R300‐treated injured kidney. Annotations based on cell‐specific markers revealed differences in various cell populations (**Figure** **3**A; Figure S3A, Supporting Information). Notably, the R300‐treated I/R group exhibited an increased presence of renal proximal TECs, a minor reduction in macrophages, and a pronounced decrease in fibroblasts compared to the IgG‐treated group (Figure 3B). Differential gene expression analysis of fibroblasts from the IgG‐treated injured kidney compared to the R300‐treated group showed significant upregulation of genes linked with collagen synthesis and immune regulation, such as SPP1, known for its profibrotic activity (Figure 3C). A similar decrease in the expression of SPP1 was identified in proximal TECs (Figure S3B, Supporting Information). Next, pathway enrichment analyses utilizing GSEA Hallmark terms highlighted several signaling pathways modulated in the context of platelets. Specifically, there was a marked downregulation of inflammatory pathways, such as inflammatory response and TNFA signaling, in the fibroblasts of the R300‐treated group. Conversely, pro‐fibrosis signals, exemplified by the collagen formation and TGF‐β pathway, were augmented. This suggested the potential influence of platelets on fibroblast activity and the overall fibrogenic process (Figure 3D). Parallel trends in pathway alterations were observed in proximal TECs, which demonstrated suppressed pro‐inflammatory pathways post‐R300 treatment (Figure S3C, Supporting Information).

An assessment of macrophage gene expression across the two treatment groups paralleled fibroblast findings, supporting evidence of the effect of platelet depletion on macrophage gene expression patterns (Figure S3D,E, Supporting Information). Exploration of cell‐cell communication unveiled robust interactions between fibroblasts and macrophages in the IgG‐treated group. In contrast, the R300‐treated group showed diminished interactions between these cell types (Figure 3E). In‐depth analysis of the signaling events between macrophages and fibroblasts in the presence of platelets led us to note THBS1 as a predominant signal modulator for both cell types (Figure 3F,G). By closely examining ligand‐receptor interactions, with an emphasis on *Thbs1*, we discovered the multiple intricate communication pathways potentially emanating from macrophages and targeting fibroblasts in a platelet‐rich environment. To confirm the hypothesis of that most THBS1 is platelet‐derived, we performed Western blot analyses to confirm the pronounced expression of THBS1 protein in platelets. In contrast, both macrophages and fibroblasts demonstrated lower expression of this protein (Figure 3H,I). These findings highlight the importance of platelet‐derived THBS1 signaling in the interaction between macrophages and fibroblasts throughout the development of I/R‐induced kidney fibrosis.

### 2.4 A Novel Platelet‐Dependent Subset Cycling M2 Macrophages Exhibits Profibrotic Characteristics

**Figure 4:**
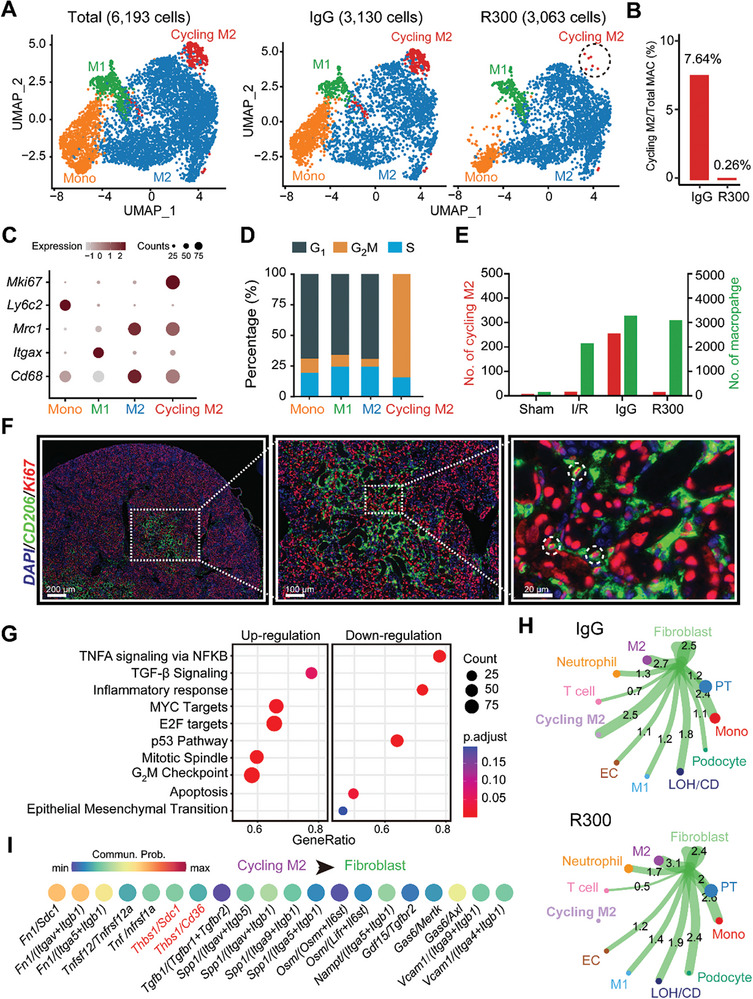
A novel platelet‐dependent subset cycling M2 macrophages exhibits profibrotic characteristics. A) UMAP projection of a total of 6193 monocyte/macrophages including 3130 cells from the IgG‐treated injured kidney, and 3063 cells from the R300‐treated injured kidney. B) The bar plot shows the percentage of cycling M2 out of the total macrophages separated by sample. C) Expression pattern of the indicated genes in all monocyte/macrophage cell types in the two samples. D) Percentage of cell cycle phases in each cell type. E) Quantification of the number of cycling M2 and M2 in mouse kidneys of the four indicated groups. F) Representative fluorescence images of cycling M2 (CD206/Ki67) in the injured kidneys in 7th day after I/R injury. G) Signaling pathways enriched in cycling M2 as compared to M2 macrophages. H) Predicted potential intercellular cell‐cell communications between fibroblasts and other cell types. I) The top 20 ligand/receptor (LR) pairs arising from cycling M2 macrophages outgoing into fibroblasts.

Given the pronounced macrophage‐fibroblast communication evident in the presence of platelets, our attention was drawn to the macrophages given their role in modulating signaling in fibroblasts. Further analyzing the previous scRNA‐seq data (Figure 3), we mapped out a UMAP projection of a cumulative of 6193 monocyte/macrophages, consisting of 3130 cells from the IgG‐treated injured kidney and 3063 cells from the R300‐treated injured kidney (**Figure** **4**A). A careful assessment of the macrophage population unveiled a distinct proliferative macrophage subpopulation we termed “cycling M2”. This subpopulation appears to be modulated by platelets, as we see a marked reduction in this subpopulation the I/R injured kidneys with R300 treatment (Figure 4B). A closer look at the gene expression within the cycling M2 macrophage subset showed that the cycling macrophages as having an M2‐like phenotype coupled with proliferative capabilities (Figure 4C). Further investigation of the cycling M2 macrophage group demonstrated a heightened mitotic activity, characterized by a notably high G_2_M phase (Figure 4D). Moreover, quantification of the cycling M2 macrophage on combined scRNA data showed that the subpopulation was minimal during the acute stage of AKI, and the subpopulation peaked during the resolution stage (Figure 4E). Immunofluorescence staining and confocal microscopy analysis confirmed the existence of the cycling M2 population that expressed both the M2 marker CD206 and the proliferation marker Ki67 in the injured kidneys at 7th post‐I/R injury (Figure 4F). To validate this, we examined a public scRNA‐seq dataset (GSE139506) and noted the presence of this subpopulation in I/R‐injured kidneys, peaking notably on the 7th day post‐surgery (Figure S4A–D, Supporting Information). Further analysis of the gene expression profile of the cycling M2 suggests it as a unique subpopulation of M2 macrophages. Beyond *Pf4* expression, this subset not only mirrors the gene signature of M2 macrophages but also exhibits a pro‐proliferative gene expression pattern (Figure S4E, Supporting Information). GSEA Hallmark term analysis of the cycling M2 subset, juxtaposed with M2 macrophages, supported these findings. The cycling M2 displayed an upregulated presence of profibrotic pathways, encompassing TGF‐β, EMT, and IL6/JAK/STAT3, along with proliferative pathways such as KRAS, E2F, and G_2_M checkpoint (Figure 4G). Exploring the intercellular communications between fibroblasts and other cell types, we noted that R300 treatment attenuated interactions between cycling M2 macrophages and fibroblasts (Figure 4H). Furthermore, we observed multiple upregulated ligand/receptor pairings arising from cycling M2 macrophages targeting fibroblasts. Within these interactions, THBS1 signaling was prominently active (Figure 4I), indicating the pivotal effect of THBS1 on modulating the cycling M2 macrophage subset and its corresponding functions. Overall, our results spotlight a novel macrophage subset termed “cycling M2”, characterized by its unique proliferative and M2‐like features.

### 2.5 Platelet Engagement Drives the Proliferation and Presence of Cycling M2 Macrophages

**Figure 5:**
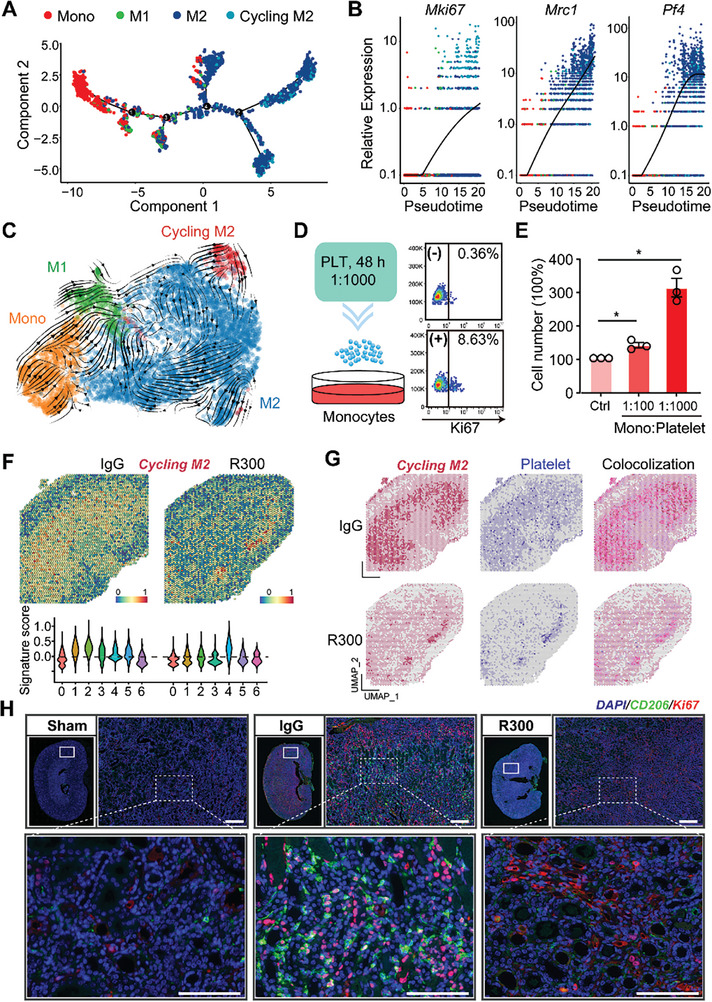
Platelet engagement drives the proliferation and presence of cycling M2 macrophages. A) Pesedoutime projection of the potential cell differentiation trajectory of the four cell types by using the R package “monocle2”. B) The relative expressions of *Mki67, Mrc1*, and *Pf4* in each cell type along pseudotime. Each dot represents an individual cell under the pesedoutime projection. C) RNA velocity shows the potential differentiation direction of the four cell types. D,E) RAW 264.7 cells were treated with different numbers of platelets for 48 h and subjected to flow cytometry for Ki67 expression D) or cell counting E), *n* = 3 for each group, the statistics were analyzed using an unpaired two‐tailed Student's t‐test, ^*^ *p* < 0.05. F) Spatial feature and violin plot of cycling M2 macrophage signature in ST spots and its relative expressions in each cluster of the IgG‐treated injured kidney as compared to the R300‐treated ones. G) The distribution of cycling M2, platelet's signature, and their colocalization in ST spots of the indicated two groups. H) Representative fluorescence images and quantification of cycling M2 in the kidneys from the IgG‐treated injured kidney and R300‐treated ones (*n* = 5 for each group). Scale bar: 50 µm.

We next examined the transcriptional factors (TFs) profile across different monocyte/macrophage types. Several TFs, mainly Jun, Maf, and Rel, were enriched in both the M2 and cycling M2 subsets (Figure S5A,B, Supporting Information). Intriguingly, the cycling M2 macrophages showcased distinct metabolic profile with an upregulation in thiamine and pyrimidine metabolism pathways (Figure S5C, Supporting Information), which are pivotal for cellular proliferation.^[^ ^28^ ^]^ Utilizing the “monocle2” R package, we traced the potential cell differentiation trajectory of the four macrophage subsets. The pseudotime projection illustrated that monocytes were the initial responders to the injured kidneys, succeeded by M1 macrophages. Notably, both the M2 macrophages and cycling M2 appear later in the temporal sequence of AKI (**Figure** **5**A). An interesting observation was the congruent expression of the *Pf4* gene with the markers for both M2 and cycling M2, reinforcing the contribution of platelets in driving the profibrotic M2 macrophages (Figure 5B). The RNA velocity analysis suggested at a potential differentiation trajectory wherein cycling M2 might differentiate into M2 macrophages (Figure 5C). To test the role of platelets in this process, we exposed RAW 264.7 monocytic cells to varying platelet counts for a period of 48 h. Subsequent cell counting and flow cytometry analysis demonstrated a heightened proliferation capability in RAW 264.7 cells in the presence of platelets, as evidenced by the expression of the proliferation marker, Ki67 (Figure 5D,E). Exploring the spatial attributes, a pronounced presence of cycling M2 macrophage signature was observed in spatial transcriptomic spots from the IgG‐treated injured kidney when compared to the R300‐treated group. Notably, these cycling M2 macrophages were found to colocalize with a platelet signature (Figure 5F,G). Immunofluorescence staining and confocal microscopy analysis further confirmed a significant reduction in the cycling M2 population in R300‐treated injured kidneys, compared to their IgG‐treated counterparts (Figure 5H). Our findings indicate that the presence and activity of this cycling M2 subset are modulated by platelets, emphasizing its role in the progression of kidney fibrosis.

### 2.6 Platelet THBS1 Antagonism Diminishes Cycling M2 Macrophage Proliferation and Alleviates Kidney Fibrosis

**Figure 6:**
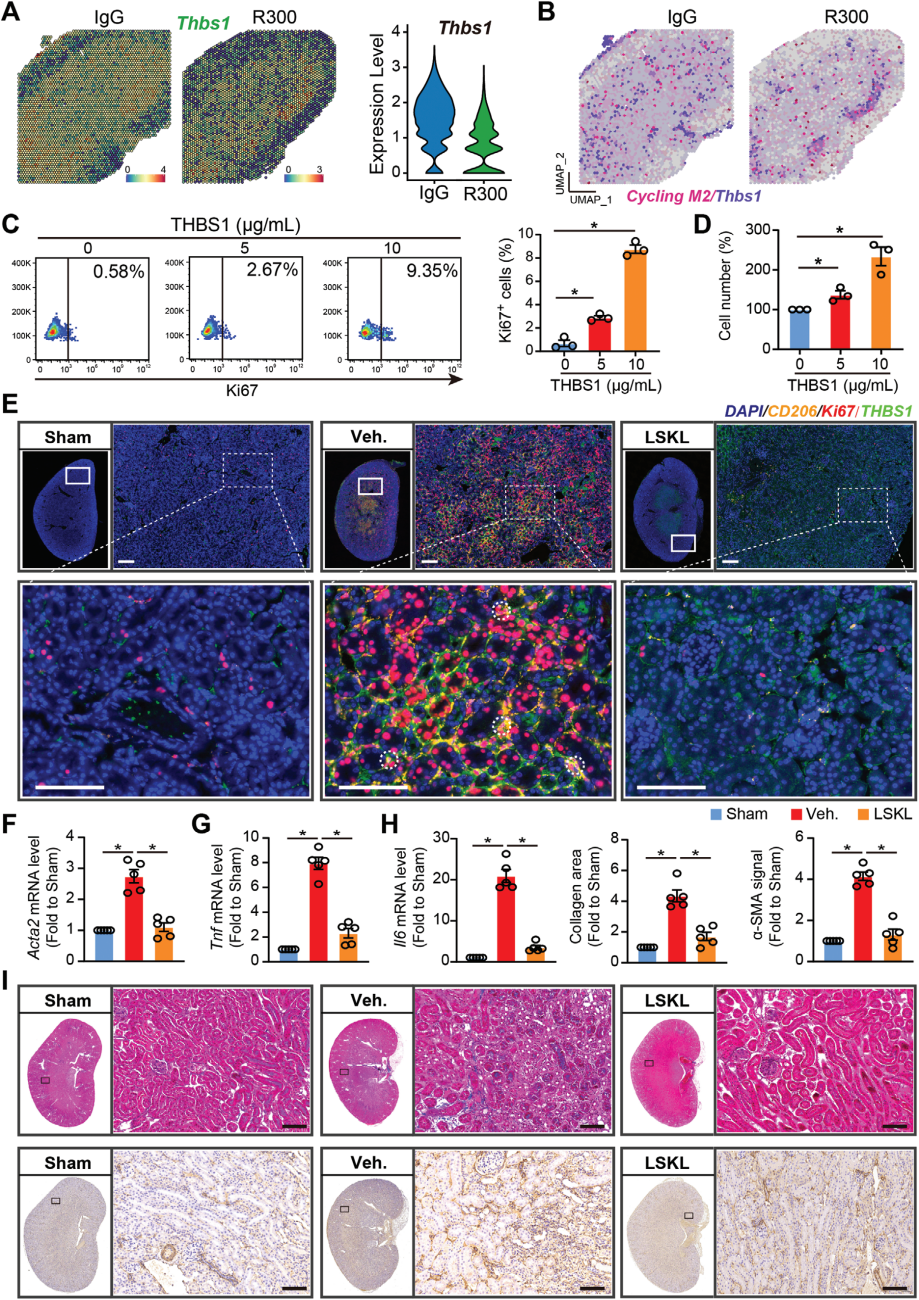
Platelet THBS1 antagonism diminishes cycling M2 proliferation and alleviates kidney fibrosis. A) Spatial feature plot of *Thbs1* in ST spots of the IgG‐treated injured kidney as compared to the R300‐treated ones and Violin plot showing its relative expression. B) Feature plot showing the colocalization of *Thbs1* and cycling M2 in ST spots of the IgG‐treated injured kidney and the R300‐treated ones. Mice received the kidney I/R injury treatment with or without THBS1 inhibitor LSKL. 7 or 21 days after the I/R injury, mice were sacrificed and kidney tissues were harvested for immunofluorescence (Day 7) or histopathology (Day 21), respectively. C,D) RAW 264.7 cells were treated with different doses of THBS1 and subjected to flow cytometry for Ki67 expression (C), or cell counting (D). E) Representative fluorescence images of CD206, Ki67, and THBS1 in the kidneys of the indicated three groups (*n* = 5 mice per group), Scale bar: 50 µm. F–H) The relative mRNA expression of *Tnf*, *Il6*, and *Acta2* were analyzed in the kidneys of the indicated three groups (*n* = 5 mice per group). I) Representative whole kidney staining images of MASSON, and IHC for α‐SMA from each group were displayed (*n* = 5 for each group), Scale bar = 50 µm. All values are presented as mean ± s.e.m. The statistics were analyzed using an unpaired two‐tailed Student's *t*‐test, ^*^ *p* < 0.05.

Upon evaluating the role of platelet‐derived THBS1 in macrophage‐fibroblast communication, the spatial transcriptomic analysis revealed an augmented expression of *Thbs1* in IgG‐treated injured kidneys as compared to R300‐treated kidneys. This upregulated expression is concurrent with the gene signature of cycling M2 macrophages, suggesting a significant colocalization (**Figure** **6**A,B). To test the role of THBS1 in this process, we exposed RAW 264.7 monocytic cells to THBS1 for a period of 48 h. Subsequent cell counting and flow cytometry analysis showed a heightened proliferation capability in RAW 264.7 cells in the presence of THBS1 (Figure 6C,D). To investigate the implications of platelet‐derived THBS1 in I/R‐induced kidney fibrosis, mice subjected to kidney IRI were administered either the LSKL peptide, a THBS1 antagonist, or treated with a vehicle and subsequently analyzed on Day 7 via immunofluorescence staining, and on Day 21 through histopathological evaluation and mRNA assessment. Whole slide kidney imaging and H&E staining demonstrated a noticeable reduction in fibrotic lesions in I/R‐operated kidneys treated with LSKL peptide when compared with the vehicle‐treated group (Figure S6A, Supporting Information). Further, immunofluorescence staining for CD206/Ki67/THBS1 exhibited suppression of both cycling M2 macrophages upon LSKL peptide treatment (Figure 6E). In agreement with the preceding observations, qRT‐PCR data showed reduced transcriptional levels of *Acta2, Tnf, Il6, Il1b, Il8, Il10, and Il12* following THBS1 inhibition (Figure 6F–H; Figure S6B, Supporting Information). The findings were validated by the reduced mRNA expressions of fibrotic markers *Acta2, Tgfb1*, as well as inflammatory cytokines *Tnf, Il6, Il1b, Il8, Il10*, and *Il12* in R300‐treated kidneys (Figure S6C,D, Supporting Information). This observation was consistent with Masson trichrome staining which demonstrated diminished collagen deposition in LSKL‐treated kidneys. Concurrently, IHC results for α‐SMA supported this finding (Figure 6I). Taken together, these observations highlight the potential of the THBS1 antagonist, LSKL peptide, in reducing kidney fibrosis and limiting M2 macrophage proliferation.

### 2.7 Deletion of *Thbs1* in Mice Shields Kidneys from Fibrotic Changes Post‐Injury

**Figure 7:**
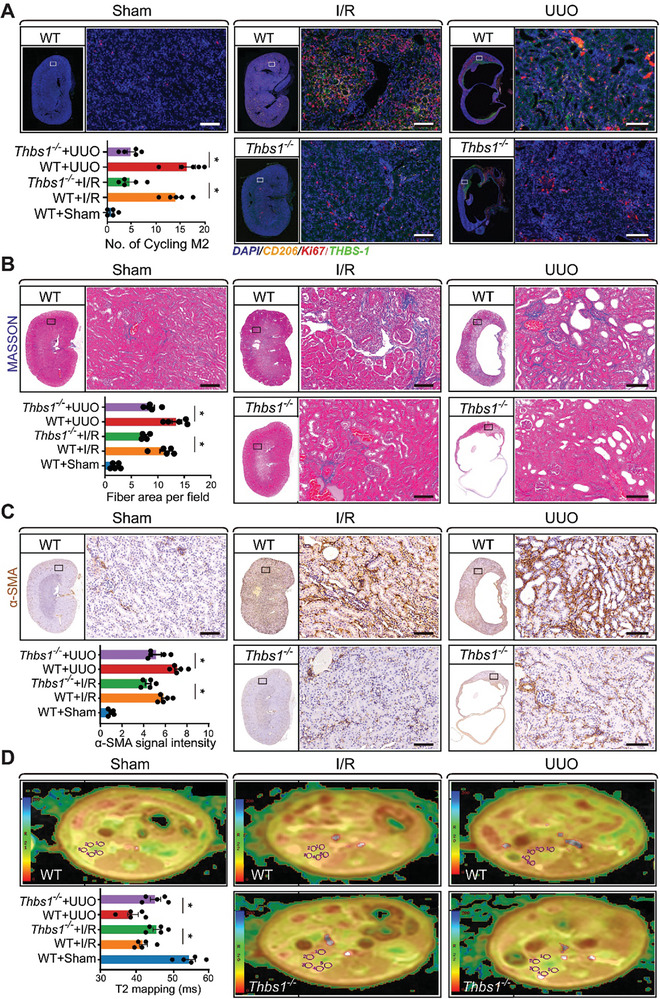
Deletion of platelet *Thbs1* in mice shields kidneys from fibrotic changes post‐I/R injury. Wild‐type or *Thbs1* knockout mice were subjected to the surgery for bilateral I/R kidney injury or unilateral ureteral obstruction (UUO) and housed for 7 days. After that, mice were sacrificed, and kidney tissues were harvested for immunofluorescence. A) Representative images and the quantifications of THBS1/CD206/Ki67 immunofluorescence (IF) signal in the kidney (*n* = 5 for each group). Wild‐type or *Thbs1* knockout mice were subjected to the surgery for bilateral I/R kidney injury or UUO and housed for 21 days and 14 days (*n* = 5 for each group), respectively. After that, all the mice first received Nuclear Magnetic Resonance Imaging (NMRI), followed by sacrificing, and kidney tissues were harvested for histopathology, Scale bar: 50 µm. B) Representative MASSON staining images and the quantifications of interstitial collagen accumulation (blue signal), Scale bar: 50 µm. C) Representative images and the quantifications of α‐SMA IHC signal in the kidney (*n* = 5 for each group). The whole pictures of the serial sections of the stained kidney were also displayed, and statistics between the indicated groups were analyzed using an unpaired two‐tailed Student's *t*‐test, and the significance was labeled with an asterisk; Scale bar: 50 µm. D) Representative NMRI images of the mouse kidney and the quantifications of T2 mapping for the kinetic motion of water molecules in the renal cortex; All values are presented as mean ± s.e.m.; *n* = 5 for each group, the statistics were analyzed using an unpaired two‐tailed Student's *t*‐test, ^*^ *p* < 0.05.

To determine the influence of platelet THBS1 on the progression of kidney fibrosis, we used both wild‐type and *Thbs1* knockout mouse models. These mice underwent surgeries inducing either bilateral I/R kidney injury or unilateral ureteral obstruction (UUO). Seven days post‐surgery, kidney tissues from these mice were analyzed using immunofluorescence techniques (Figure S7A, Supporting Information). Remarkably, quantification of THBS1/CD206/Ki67 immunofluorescence highlighted a notable decrease in cycling M2 macrophages in the kidneys of *Thbs1* knockout mice, irrespective of whether the injury was due to I/R or UUO, compared to their wild‐type counterparts (**Figure** **7**A). In a prolonged study, wild‐type and *Thbs1* knockout mice subjected to bilateral I/R kidney injury were observed for 21 days, while those subjected to UUO were observed for 14 days. Before sacrificing these mice for tissue harvesting, nuclear magnetic resonance imaging (NMRI) was performed. Histopathological evaluations, including H&E staining, indicated that *Thbs1* knockout mice exhibited diminished fibrotic lesions following either I/R kidney injury or UUO, as compared to the wild‐type mice (Figure S7B, Supporting Information). This observation was further supported by Masson's trichrome staining data, which demonstrated reduced interstitial collagen deposition in the kidneys of the *Thbs1* knockout mice for both injury models (Figure 7B). Furthermore, the α‐SMA IHC signal quantifications in the kidney tissues indicated a marked reduction in α‐SMA expression in the *Thbs1* knockout mice after injury (Figure 7C). qRT‐PCR data demonstrated reduced mRNA levels of *Acta2 and Tgfb1* following *Thbs1* deletion (Figure S7C, Supporting Information). To evaluate the extent of fibrosis in more detail, we utilized Nuclear Magnetic Resonance Imaging (NMRI). This non‐invasive technique, specifically the T2 mapping analysis, measures the transverse relaxation time in the kidney. The rationale behind this is that T2 values decline in fibrotic kidneys due to the restricted kinetics of water molecules.^[^ ^29^ ^]^ As anticipated, the NMRI images alongside the T2 mapping quantifications showcased that, in contrast to healthy kidneys, fibrotic kidneys possess diminished water content, affirming the efficacy of T2 as a reliable marker for kidney fibrosis (Figure 7D). Conclusively, these results suggest that the genetic ablation of *Thbs1* offers substantial protection against kidney fibrosis.

## 3 Discussion

AKI represents a complex clinical syndrome with multifactorial etiologies and significant morbidity, often culminating in CKD and posing considerable challenges in therapeutic management. In our current study delving into the molecular dynamics following ischemia/reperfusion (IR) injury, we utilized the cutting‐edge capabilities of single‐cell and spatial transcriptomics to reveal an underappreciated role of platelets as a pivotal risk factor in the progression of AKI to kidney fibrosis. Our study has demonstrated the intricate landscape of cell‐cell interactions within the kidney following an IRI‐induced AKI. In addition to platelets, we have also identified the cycling M2 macrophage population, a unique macrophage subset displaying an M2‐like phenotype. This subset not only showcased pronounced profibrotic markers but also exhibited a notable proliferative capacity. We have found that this population is expanded by platelets in the context of AKI to CKD transition and attenuated by targeting platelets, suggesting that this population plays a role in mediating the fibrotic process.

A notable finding from our data is the pivotal role of platelets in dictating kidney injury severity as evidenced by the positive correlation between platelet counts and serum creatinine levels (SCr). This association is in line with findings from an expansive study that analyzed 10859 ICU patients with AKI from the Multiparameter Intelligent Monitoring in Intensive Care (MIMIC) Database III. This prior study discerned a distinct U‐shaped correlation between mortality and the platelet‐to‐lymphocyte ratio (PLR). The results suggest that patients with abnormally high or low PLRs are alarmingly predisposed to a heightened mortality risk.^[^ ^30^ ^]^ Similarly, another study involving 1938 adult septic patients from the MIMIC Database IV identified a direct association between an elevated neutrophils‐to‐lymphocytes and platelets (N/LP) ratio and increased susceptibility to sepsis‐induced AKI and its subsequent severity.^[^ ^31^ ^]^ On the contrary, research encompassing 4217 adult patients undergoing coronary artery bypass grafting unveiled an inverse relationship: those exhibiting lower nadir platelet counts were at a heightened risk of developing AKI.^[^ ^32^ ^]^ The diversity of these findings suggest that the relationship between platelets and AKI remains an active area of investigation and warrants further efforts to elucidate the relationship between the two.

Although historically confined to the realms of hemostasis and thrombosis, there is increasing interest in platelets due to their dynamic roles as inflammatory mediators and function as potent immune modulators. Platelets have been noted to play in the role in pathogeneses of diseases as diverse as atherosclerosis, rheumatoid arthritis, and cancer, where they play instrumental roles in tumorigenesis and metastasis.^[^ ^33^ ^]^ Given their role in multiple functions platelets emerge as a promising therapeutic target. For instance, aspirin has been shown to mitigate tissue damage in the context of cardiac IRI through reducing platelet aggregation.^[^ ^34^ ^]^ Furthermore, the preventive efficacy of aspirin was evident when its early administration in post‐coronary bypass surgery scenarios led to a significant 74% reduction in renal failure episodes.^[^ ^35^ ^]^ Likewise, Clopidogrel, an anti‐platelet agent, has demonstrated remarkable efficacy in ameliorating IRI purportedly through enhancing the renal antioxidant capacity, a pivotal factor in countering IRI‐induced oxidative damage.^[^ ^36^ ^]^ Our observations have unveiled that employing antibody‐mediated targeted depletion of platelets can confer significant protection against kidney injury and fibrosis in murine models post‐IRI. Given that AKI is a frequent problem in the ICU with a dearth of pharmacologic therapeutic options, our findings may provide a viable therapeutic avenue for the treatment of AKI to CKD transition through targeting of platelet‐initiated signaling. This research highlights the critical interplay between platelets and AKI to CKD transition. Our findings, with support from other key studies, suggest potential in platelet‐targeted therapeutic strategies. As we deepen our understanding, these insights may guide future interventions for CKD caused by IRI‐induced AKI, potentially transforming clinical approaches.

Prior studies have demonstrated the effect of THBS1 in modulating inflammation and kidney injury in the context of AKI.^[^ ^24^, ^25^, ^26^, ^27^ ^]^ Notably, it has been previously demonstrated that THBS1 null mice are protected from renal I/R injury.^[^ ^23^ ^]^ Additionally, other studies have also pointed to the role THBS1 plays in mediating fibrosis in CKD. Within our study, we have found that platelet‐derived THBS (TSP‐1), which emerged as a master regulator, orchestrates intricate interactions with fibroblasts and macrophages in the AKI to CKD transition. These interactions seem to significantly impact the fibrotic landscape within the kidney post‐IR injury. Delving deeper and in line with prior findings, our work demonstrated the direct impact of THBS1 on fibrosis, specifically its role in promoting the production of extracellular matrix (ECM). Moreover, our studies in murine models revealed that antagonizing THBS1 or its genetic deletion led to a marked reduction in the emergence of the M2‐like macrophage phenotype and kidney fibrosis. THBS1 is rapidly mobilized from platelets to the sites of tissue injury, where it becomes a critical regulator, directing a plethora of signaling pathways vital to the body's response. It plays a pivotal role in mediating the activation of latent TGF‐beta, an essential process in tissue repair and fibrosis. THBS1 also modulates inflammation, endoplasmic reticulum stress, coordinates extracellular matrix organization, and promotes angiogenesis, emphasizing its multifaceted importance.^[^ ^37^ ^]^ Beyond modulation of multiple biological pathways, THBS1 is also a regulator of immune cells. Macrophages, NK cells, and T cells, are recruited to sites of injury and are modulated by THBS1. Intriguingly, the presence of multiple THBS1 receptors on these cells permits a vast array of distinct immune responses, leading to a nuanced modulation of inflammation within affected tissues.

THBS1 is well‐known for its role as an activator of latent TGF‐β1 (LTGF‐beta1), a potent mediator of fibrotic kidney disease.^[^ ^38^ ^]^ Histological comparisons between *Tgfb1* ^−/−^ and *Thbs1* ^−/−^ mice were found to be similar across several organ systems, with both lines sharing features such as diffuse inflammation and an indistinct kidney cortico‐medullary junction. *Thbs1* ^−/−^ mice were found to produce low levels of active TGF‐β1; however, treatment of these mice with a THBS1‐derived peptide was able to revert lung and pancreatic abnormalities.^[^ ^39^ ^]^ Thus, several studies have implicated THBS1 as a mediator of tubulointerstitial fibrosis due to its role in activating TGF‐β.^[^ ^40^ ^]^ As global inhibitors of TGF‐β have failed to show efficacy, methods specifically targeting pathogenic TGF‐β signaling through THBS1 have been of high interest ^[^ ^41^ ^]^ Treatment of Akita mice undergoing uninephrectomy with the LSKL peptide, an antagonist of THBS1, drastically reduced diabetic tubulointerstitial injury and fibrosis by inhibiting TGF‐β activation.^[^ ^42^ ^]^ In a model of mesangial proliferative glomerulonephritis, continuous intravenous infusion of either LSKL or AAWSHW both THBS1 blocking peptides resulted in reduced active TGF‐β and ECM deposition in the glomeruli. Notably, while peptide‐mediated blocking of THBS1 decreased renal fibrosis by preventing TGF‐β activation, it did not affect mesangial cell proliferation, nor did it decrease monocyte/macrophage infiltration.^[^ ^39^ ^]^ As THBS1 is known to promote both mesangial cell proliferation and monocyte recruitment, THBS1‐driven renal fibrosis likely involves mechanisms beyond the activation of latent TGF‐β.^[^ ^43^ ^]^

Our study identified THBS1 as a potential mediator of cellular cross‐talk between macrophages and fibroblasts. This finding aligns with existing literature that supports the concept of THBS1 playing a significant role in cellular interactions. When human endothelial cells (ECs) were exposed to apoptotic cells in co‐culture with human primary macrophages, the ECs produced increased amounts of THBS1 and triggered pro‐inflammatory and phagocytotic responses.^[^ ^44^ ^]^ Apoptotic fibroblasts upregulated THBS1 expression and interacted with macrophages to promote phagocytosis through upregulated interactions between the fibroblast CD36/THBS1 complex and macrophage αvβ3.^[^ ^45^ ^]^ Co‐cultures of adipocytes with M1 or M2c macrophages induced the production and secretion of THBS1. IL‐10 knock‐out with siRNA in M2c cells reduced THBS1 expression, suggesting that IL‐10 plays a regulatory role in THBS1 production.^[^ ^46^ ^]^ Consistent with these observations, our study highlights the importance of THBS1 as not only a chemotactic cytokine for monocytes but also as a polarizing cytokine for a novel subset of pro‐fibrotic macrophages with a strong proliferative ability. In contrast to our findings,^[^ ^27^, ^47^, ^48^ ^]^ other studies in endothelial and epithelial cells have suggested that THBS1 attenuates self‐renewal and proliferation suggesting that there may be other factors at play. This difference could potentially be attributed to variations in cellular signaling pathways between immune cells and epithelial/endothelial cells. Similarly, THBS1 has been reported to induce cytotoxicity in macrophages, such as RAW 264.7 cells, in the context of LPS exposure;^[^ ^49^ ^]^ however, our research did not observe this effect. This discrepancy underscores the complex nature of THBS1's interactions with cellular mechanisms, which may significantly vary depending on experimental conditions, cell type‐specific factors, or the activation state of the cells. Nevertheless, these aspects require further investigation, which was beyond the scope of our current study.

This research investigating the cell interactions and the molecular dynamics of AKI to CKD transition in the context of IR injury also has limitations. A limitation of our current study is inherent in the utilization of omics technologies. Although through our single cell sequencing and spatial transcriptomics, we have identified multiple signal pathways of interest, these identified pathways are identified only by the presence of genes in the omics data and does not necessarily indicate significance in cell biology. This requires further validation of the identified pathways through specific experiments investigating the signaling mechanisms and expression of the signaling molecules at play in depth. Despite this, our data does suggest that immune cell populations, in particular, the cycling M2 macrophage population, is modulated by platelets and that depletion of platelets and that associated pro‐inflammatory and fibrotic pathways may be modulated by the presence and absence of platelets in AKI to CKD transition. Additionally, given the role of THBS1 in modulating various angiogenic pathways, consideration of its effect in blood flow in AKI to CKD transition is warranted. Studies have indicated that THBS1 is able to modulates levels of VEGF, eNOS, nitric oxide, cGMP, and PKG kinase.^[^ ^50^, ^51^, ^52^, ^53^ ^]^ Indeed, in CKD, renal blood flow has been shown to be significantly reduced.^[^ ^54^ ^]^ This phenomenon in addition to THBS1's role in mediating fibrotic pathways may contribute to the progression of AKI to CKD. Investigation of this phenomenon was beyond the scope of our current study but warrants further study. Lastly, THBS1 is readily secreted by multiple cell types in the kidney including endothelial, epithelial, and immune cells. Although depletion of platelet THBS1 via R300 antibody does reduce the overall level of THBS1 present, this does not affect the secretion of THBS1 from other cell types. Despite this limitation, we do still note that depletion of platelet THBS1 is able to attenuate AKI to CKD transition.

Our investigation spotlighted a unique cycling M2 macrophage population, characterized by both its strong profibrotic activity and proliferative potential. Therapies targeting this novel subset of cells could yield tremendous benefits for current treatments of AKI/CKD. By controlling this macrophage subset, we observed a significant mitigation of kidney fibrosis. Other studies have provided additional evidence of the contribution of proliferating tissue‐resident macrophages in disease states in line with findings such as in obesity‐associated chronic inflammation and atherosclerosis, emphasizing the predominance of host‐derived over bone marrow‐derived macrophages.^[^ ^55^, ^56^ ^]^ Further, in IRI models, resident kidney macrophages shifted their transcriptome to resemble neonatal kidney macrophages and heightened Wnt/b‐catenin signaling, a crucial factor for kidney repair and CKD.^[^ ^57^ ^]^ Likewise, the origin and lineage variance of these cycling M2 macrophages has been paralleled in other contexts, most notably with tumor‐associated macrophages (TAMs), which are known for their immunosuppressive M2 profile and implication in extracellular matrix remodeling within the tumor microenvironment.^[^ ^58^, ^59^ ^]^ In sum, we have found that the deletion of platelet THBS1 was observed to significantly suppress kidney fibrosis by targeting the cycling M2 macrophage population, suggesting its potential as a therapeutic target in AKI scenarios. Evident from IRI and UUO models, THBS1 deletion consistently culminated in diminished cycling M2 macrophages and fibrosis reduction, indicating its pivotal role in kidney fibrosis across various inflammatory milieus. The therapeutic promise of LSKL, a THBS1 peptide antagonist, has been demonstrated in multiple fibrotic diseases, ranging from liver atrophy to cardiac fibrosis, emphasizing its capacity to counteract TGF‐β1 activation.^[^ ^60^, ^61^ ^]^ Despite its potential, there are concerns about the long‐term ramifications of global TGF‐β inhibition. Given the important homeostatic functions of TGF‐β, further research into the potential side effects of LSKL is needed.

## 4 Conclusion

Although we have identified the role of platelets and THBS1 in modulating this cycling M2 population and subsequent downstream effects on fibrosis, further investigation into the specific signaling pathways behind this process are warranted. Our findings taken together have highlighted the multifaceted roles of platelets and THBS1, and their interplay in IR‐induced kidney injury, suggesting that targeting this pathway may be a potential therapeutic strategy to address renal fibrosis.

## 5 Experimental Section

### Clinical Information and Ethical Approval

A total of 102 patients with diverse cardiovascular diseases including 63 cases with Ventricular Septal Defect (VSD), 9 cases with Tetralogy of Fallot (ToF), 16 cases with Atrial Septal Defect (ASD), 6 cases with Coarctation of Aorta (CoA), and 8 cases with Transposition of the Great Arteries (TGA) were recruited in the current study. All the patients previously received the operation of extracorporeal circulation during cardiovascular surgery, which is considered a common risk factor for human kidney ischemia‐reperfusion injury. The information on gender, age, weight, disease type, platelet numbers, and serum creatinine levels of the patients was collected. The criteria to categorize AKI was based on the guidelines of kidney disease: Improving Global Outcomes (KDIGO), 2012.^[^ ^62^ ^]^ Participants for this study were recruited exclusively from the Soochow University community in China. In this study, all procedures involving human subjects were carried out in accordance with the ethical standards of the Institutional Review Board of the Children's Hospital of Soochow University (IRB number: 2022CS059). Informed consent was obtained from all individual participants included in the study. To protect participants' privacy and confidentiality, all identifying information was anonymized and securely stored. Data access was restricted to the research team members directly involved in data analysis. Detailed information was displayed in the online Tables S1 and S2 (Supporting Information).

### Cell Culture

RAW 264.7 and 3T3‐L1 cells were obtained from the American Type Culture Collection (ATCC) and were cultured in RPMI1640 medium (Gibco) containing 10% fetal bovine serum (FBS, Gibco) at 37 °C in an incubator with a humidified atmosphere of 5% O_2_ and 5% CO_2_. For thrombospondin 1 treatment, RAW 264.7 cells were treated with diverse doses of thrombospondin 1 (HY‐P70725, MCE) for 48 h. For platelet treatment, the cultured RAW 264.7 cells were treated with different numbers of platelets for 48 h that were obtained from mice's peripheral blood according to our previous study.^[^ ^63^ ^]^ After that, the cells were collected for cell proliferation and gene expression analysis.

### Animal Studies

*Thbs1* knockout mice (Strain No.: NM‐KO‐226309) were purchased from Shanghai Model Organisms Center, Inc. (Shanghai, China), and housed in a pathogen‐free environment, a temperature of 22 °C and relative humidity of 50%–60%. The Institutional Review Board of the Children's Hospital of Soochow University approved the animal studies (IACUC number: SUDA20200824A02). In compliance with IACUC guidelines, this study protocol, including methods for humane handling, pain relief, and euthanasia of mice, had been meticulously detailed in the Methods section. For humane handling, researchers were trained in accordance with the guidelines to ensure minimal stress to the animals. Pain relief was managed through as per the approved protocol. Euthanasia was performed using Carbon dioxide (CO_2_) overdose, considered the most humane method available and in line with the guidelines. Bilateral I/R‐induced kidney injury surgery on mice was performed as previously. Briefly, 4 to 6‐week‐old male C57BL/6J mice were intraperitoneally anesthetized with 1% (v/w) chloral hydrate. It was used male mice based on evidence that males were more susceptible to renal ischemic injury,^[^ ^64^ ^]^ the primary focus of this study. For ischemia induction, the renal pedicles were clamped with a clip for 45 min through the incision at the flank and were then followed by reperfusion by clip removal according to the previous study.^[^ ^65^ ^]^ Incisions were sutured under sterile surgical conditions, and the mice were transferred to the standard housing environment for recovery. Mouse kidney and blood samples were collected for analysis of serum creatinine (Scr), blood urea nitrogen (BUN), and histopathology at the needed times. For platelet depletion, to avoid uncontrollable bleeding, mice were intravenously injected with a dose of 4 µg kg^−1^ platelet‐depleting antibody R300 (Emfret Analytics) through the tail vein 48 h after the I/R injury.^[^ ^66^ ^]^ On the 7th or 21st day after the surgery, mice were sacrificed, and the kidneys were harvested for single‐cell spatial transcriptomics or histopathological experiments, respectively. The dose of the platelet‐depleting antibody was established through initial dose‐response studies aimed at maximizing platelet clearance while minimizing adverse effects. The antibody targets specific antigens on the platelet surface, facilitating their removal from circulation. Administered post‐injury, this intervention was designed to elucidate platelets' role in the inflammation and repair mechanisms following tissue damage. For LSKL treatment, mice intraperitoneally received LSKL (P1164, Selleck) with 5 mg kg^−1^ every other day for a total of 5 times or vehicle alone. For UUO surgery, the unilateral ureter of wild‐type C57BL/6J or *Thbs1* knockout mice was exposed and ligated with an absorbable suture under anaesthetization and sterile conditions. The mice were recovered and housed in the standard environment as above.

### Nuclear Magnetic Resonance Imaging (NMRI)

NMRI was conducted on mouse kidneys by using a GE 3T MRI Discovery MR750 machine (GE HealthCare Technologies Inc., Chicago, Illinois, US) equitable with a coil for small animals according to previous studies.^[^ ^67^ ^]^ Animals were anesthetized by 2% isoflurane, and placed in the supine position, letting the abdomen in the center of the coil. For functional T2 imaging, the kidney volume was quantified by using a T2‐weighted multisection imaging with the following parameters: time of repetition per time of echo (TR/TE): 2000/30, field of view: 35 × 35 mm^2^, matrix: 128 × 128, section thickness: 1 mm, diffusion time: 20 ms; gradient pulse duration: 5 ms. T2 was averaged by randomly selecting five 1 × 1 mm areas of the renal cortex using MRI Discovery MR750 analysis systems. To improve the robustness of our data and ensure investigator blinding, experiments were performed with the researchers unaware of the treatment assignments until the conclusion of data analysis. Randomization of samples and standardized protocols were strictly followed to reduce bias. Moreover, we incorporated biological and technical replicates to bolster the reliability of our findings.

### qRT‐PCR

Total RNA was extracted using Trizol Reagent (Invitrogen). RNA was reverse transcribed into cDNA using a High‐Capacity cDNA Reverse Transcription Kit (4 368 814, Applied Biosystems). Quantitative PCR was performed using the qPCR SYBR Green Master Mix (Takara) and analyzed using a LightCycler 96 real‐time system (Roche Diagnostics). Relative quantification was calculated using the 2^ΔΔCt^ method and normalized to GAPDH. The primer sequences are listed in Table S3 (Supporting Information).

### Immunoblot

The cell lysate was obtained by homogenizing using a protease and phosphatase inhibitor cocktail (0 469 311 6001, Roche)‐contained RIPA lysis buffer (R0010, Solarbio, Beijing, China). The protein concentrations were measured by using BCA assay (Solarbio). Protein samples were separated using 10% SDS page gels and transferred onto a PVDF membrane (R1SB07718, Millipore). After blocking using 5% skimmed milk for 1 h at room temperature, the membrane was incubated with primary antibody THPS1 (37 879, Cell Signaling), or β‐actin (4970, Cell Signaling) at 4 °C overnight and then with rabbit HRP‐linked antibody (7074, Cell Signaling) for 1 h at room temperature. The membrane was washed three times with 1 × PBST between incubations. Proteins were visualized by enhanced chemiluminescence (ECL, 32 209, Thermo Fisher Scientific). The detailed information and validation of antibodies was listed in Table S4 (Supporting Information).

### Histopathological Analysis and Immunofluorescence

Mouse kidneys were collected and fixed in 4% formaldehyde (PFA), followed by dehydration using an ascending graded series of ethanol, and paraffinization. For kidney fibrosis detection, 5‐µm thick tissue sections were prepared and stained with hematoxylin and eosin (H&E) solution or Masson Trichrome Stain (Masson) Kit (HT15, Sigma–Aldrich) according to the instructions. Images were visualized and captured with a digital camera‐equipped microscope (Olympus). The blue area of Masson staining was calculated for fibrosis evaluation based on the 10 randomly selected interstitium using ImageJ software (National Institutes of Health). For IHC staining, tissue sections were fixed with 4% PFA overnight and washed 3 times with PBS. After that, tissue sections were dewaxed in xylene, rehydrated using a descending graded series of ethanol, and incubated in EDTA buffer at 95 °C for 30 min to induce epitope retrieval. Tissue sections were then blocked with 5% goat serum and incubated with alpha smooth muscle Actin (α‐SMA) (ab124964, Abcam). For immunofluorescence, 5 µm thickness tissue sections were mounted onto poly‐L‐lysine coated slides, permeabilized, and blocked with 1% BSA containing 0.3% Triton‐X 100 for 1 h. Sections were incubated with primary antibodies against CD68 (ab283654, Abcam), CD206 (ab64693, Abcam), Ki67 (ab16667, Abcam), Thrombospondin 1 (THBS1) (ab267388, Abcam), and PF4 (ab303494, Abcam) overnight at 4 °C followed by incubation with secondary antibodies and DAPI. The sections were washed 3 times with 1 × pre‐chilled PBS between incubations and covered with coverslips for visualization with a digital camera‐equipped microscope (Olympus).

### Flow Cytometry

For analysis of the monocyte/macrophage in mouse kidneys, the kidneys were harvested and enzymatically digested to obtain single‐cell suspensions. Cell suspensions were treated with red blood cell lysis buffer (Beyotime) to deplete red blood cells, followed by washing with 1×pre‐chilled PBS. For RAW 264.7 cells, cells were collected after treatment and washed twice with pre‐chilled PBS and resuspended to the single‐cell suspension. The cells were subsequently stained with different antibodies including anti‐Ki67‐PerCP/Cy5.5 (151 222, Biolegend). After being stained with fluorescently labeled antibodies, the cells were washed in PBS. Flow cytometry data were acquired by a BD FACSCanto II flow cytometer using the FlowJo software (v10, BD Bioscience).

### 10x Genomics Sample Processing and cDNA Library Preparation

To prepare the renal single‐cell suspension, freshly collected mouse kidneys were rinsed with RPMI1640 (Gibco), minced into 3–5 mm3 pieces under sterile conditions, and then enzymatically digested by using Multi Tissue Dissociation Kits (130‐110‐204, Miltenyi Biotec) for 15–30 min under rotation at 250 rpm min^−1^. After the filtration and centrifugation, the cell pellet was collected, resuspended in 1 mL RBC lysis buffer (Sigma) to remove RBCs, and then washed twice with RPMI1640. Cell number, viability, and the percentage of cell doublet were assessed, and dead cells were removed using magnetic bead separation with a Dead Cell Removal Kit (130‐090‐101, Miltenyi Biotec) if needed. At least 20000 cells per sample were applied to a single‐cell master mix with lysis buffer and reverse transcription reagents, following the User Guide of 10X Genomics Chromium Single Cell 3′ Reagent Kits (10X Genomics). The libraries were subjected to high‐throughput sequencing on a NovaSeq6000 platform.

### Single‐Cell RNA‐Seq Analysis

Single‐cell expression data were generated from FASTQ files, which were processed by using Cell Ranger software (version 3.1.0, 10X Genomics). The analysis began with the removal of batch effects between samples using the R package “harmony” following the pipeline (http://satijalab.org/seurat/). Quality control was performed to exclude cells with fewer than 200 genes or with mitochondrial gene percentages over 20%. Then, the dataset was normalized using the NormalizeData command and scaled using the ScaleData command in the Seurat (version 4.3.0). For unsupervised clustering, PCA analysis was performed using the RunPCA command. Dimensionality reduction was performed using the RunUMAP command and visualization of cell clustering by Uniform Manifold Approximation and Projection (UMAP). The FindAllMarkers function was used to determine cluster‐specific gene markers. Cell annotation was performed by mapping the marker genes of each cluster to canonical kidney cell‐type marker genes from previous studies.^[^ ^65^ ^]^ Cell communications were analyzed by calculating the ligand and receptor (LR) pairs using the “CellChat” package. For Gene Set Enrichment Analysis, the differentially expressed genes (DEGs) between groups were analyzed using the FindMarkers function. GSEA analysis was performed using the R package “fgsea” (1.24.0) by mapping genes to the murine reference database org.Mm.eg.db (3.16.0).

### Spatial Transcriptomics

Fresh frozen mouse kidneys were embedded in OCT (Tissue‐Tek, 458, SAKURA) for cryosectioning. Cryosection was placed in the capture area of the Visium Spatial Gene Expression slide (10X Genomics). Tissue sections were fixed with pre‐chilled methanol, stained with hematoxylin (S3309, Dako) and eosin (CS701, Dako) solution, and were visualized under brightfield by a Leica DMI8 whole‐slide scanner. The tissue was then permeabilized to release mRNA from the cells. mRNA was captured by probes on the Visum spatial gene expression slides (PN‐1000184,10X Genomics) for reverse transcription. The captured mRNA was reversely transcribed to cDNA, spatially barcoded, and amplified, and subjected to libraries construction using the Visum spatial Library construction kit (PN‐1000184,10X Genomics). The constructed library was sequenced with an Illumina Novaseq6000 sequencer with a sequencing depth of at least 100 000 reads per spot (performed by CapitalBio Technology, Beijing).

### Spatial Transcriptomics Analysis

After sequencing, tissue images, and the related FASTQ files were processed and aligned using the software SpaceRanger (v2.0.1, 10X Genomics) and the mouse reference genome GRCm39. The Space Ranger analysis pipelines were performed to generate an ST spots matrix with the input tissue image and the FASTQ sequencing data. The count matrices were processed to filter out low‐quality spots and poorly expressed genes. For quality control, spot with less than 200 genes was excluded from further analysis. Normalization across spots was performed with the SCTransform function (0.3.5), and anchors between the spatial transcriptomics spots were identified using the R package “Seurat” (v4.3.0) in R (v4.2.2). Dimension reduction and clustering were performed using PCA. The first 20 PCs were used to generate clusters. For scoring on the specific cell type, the marker gene for each cell type was selected as the signature gene to perform the AddModuleScore function with default parameters in Seurat. For spatial gene/feature colocalization, the interest genes or features were inferred and the FeaturePlot function in Seurat was used with the parameter “blend” = Ture. Gene Ontology biological processes were analyzed using the R package clusterProfiler. Pathway analyses were performed using GSEA.

### Statistical Analysis

Data distribution was normalized by Kolmogorov‐Smirnov test. The normalized data were shown as the mean±s.e.m., and statistical significance was determined using a two‐tailed Student's *t*‐test between two groups using Prism (v.11.0, GraphPad). The correlation between platelets counts and Scr levels, and AKI condition were analyzed by Chi‐square or Spearman's correlation test as appropriate. A *p*‐value less than 0.05 was considered to be statistically significant.

## Conflict of Interest

The authors declare no conflict of interest.

## Author Contributions

J. L. B. Z. and Q. C. contributed equally to this work as the first author. J.L. B. Z. and Q.C. performed single‐cell and spatial experiments and data analysis. Z. G. and L.K.C. performed animal experiments. Y.Z. and Z.G. performed a large part of cell and immunoblot experiments and analyzed the data. Y.M.M., L.W., and S.W.H. performed pathological staining and FACS experiments and helped with data analysis. Q.C., X.C. and Q.W.X. assisted with *Thbs1* knock‐out mice breeding and animal experiments. Q.C., and F.J.G. helped with manuscript preparation. J.L. C.H.C. and X.M.Y. conceived the project. J.L. and X.M.Y. directed the experiment. J.L., D.C.Y. and C.H.C. wrote the manuscript.

## Supporting information

Supporting Information

## Data Availability

The data that support the findings of this study are available from the corresponding author upon reasonable request.
