# Supplementary material for: Single‐Cell Spatial Transcriptomics Unveils Platelet‐Fueled Cycling Macrophages for Kidney Fibrosis
Source: Adv Sci (Weinh). 2024 Jun 5;11(29):2308505. doi: 10.1002/advs.202308505 (PMC11304276; doi:10.1002/advs.202308505)
Supplement: Supplementary file 1 — Supporting Information [file ADVS-11-2308505-s001.docx]

Supporting Information

Single-Cell Spatial Transcriptomics Unveils Platelet-Fueled Cycling Macrophages for Kidney Fibrosis

Jun Liu*, Bo Zheng, Qingya Cui, Yu Zhu, Likai Chu, Zhi Geng, Yiming Mao, Lin Wan, Xu Cao, Qianwei Xiong, Fujia Guo, David C Yang, Ssu-Wei Hsu, Ching-Hsien Chen*, and Xiangming Yan*


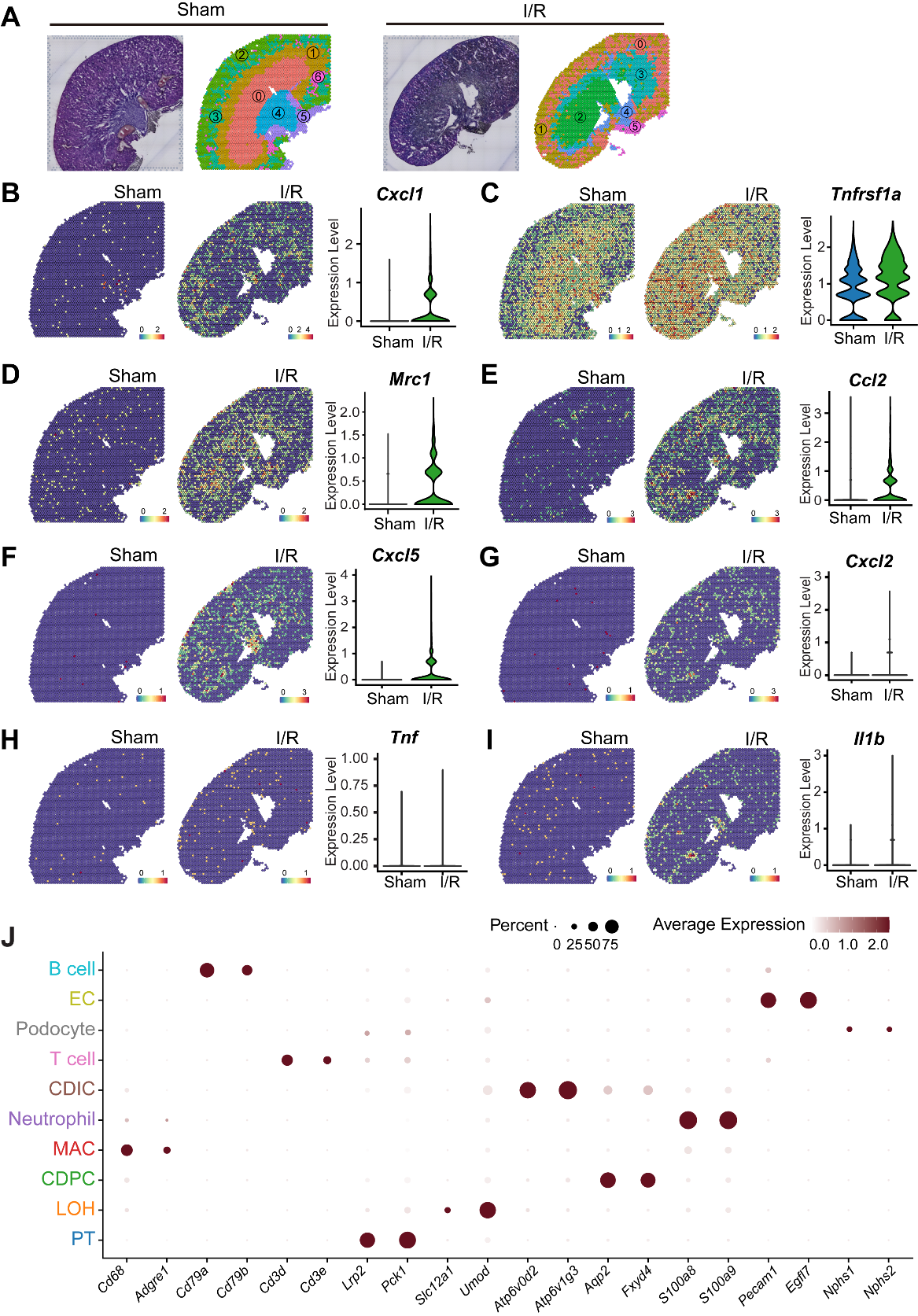


**Figure S1.** Single-cell spatial transcriptomics reveals platelet is a risk factor for kidney fibrosis. A) Hematoxylin and eosin (H&E) staining of kidney sections and unbiased clustering of the spatial transcriptome (ST) spots of sham and I/R-stimulated kidneys. B-I) Spatial feature plots and Violin plots show gene expression comparison of *Cxcl1* (B), *Tnfrsf1a* (C), *Mrc1* (D), *Ccl2* (E), *Cxcl5* (F), *Cxcl2* (G), *Tnf* (H), *Il1b* (I) in ST spots of sham or I/R- stimulated kidneys. J) The dot plot shows expressions of two representative marker genes for each cell type.


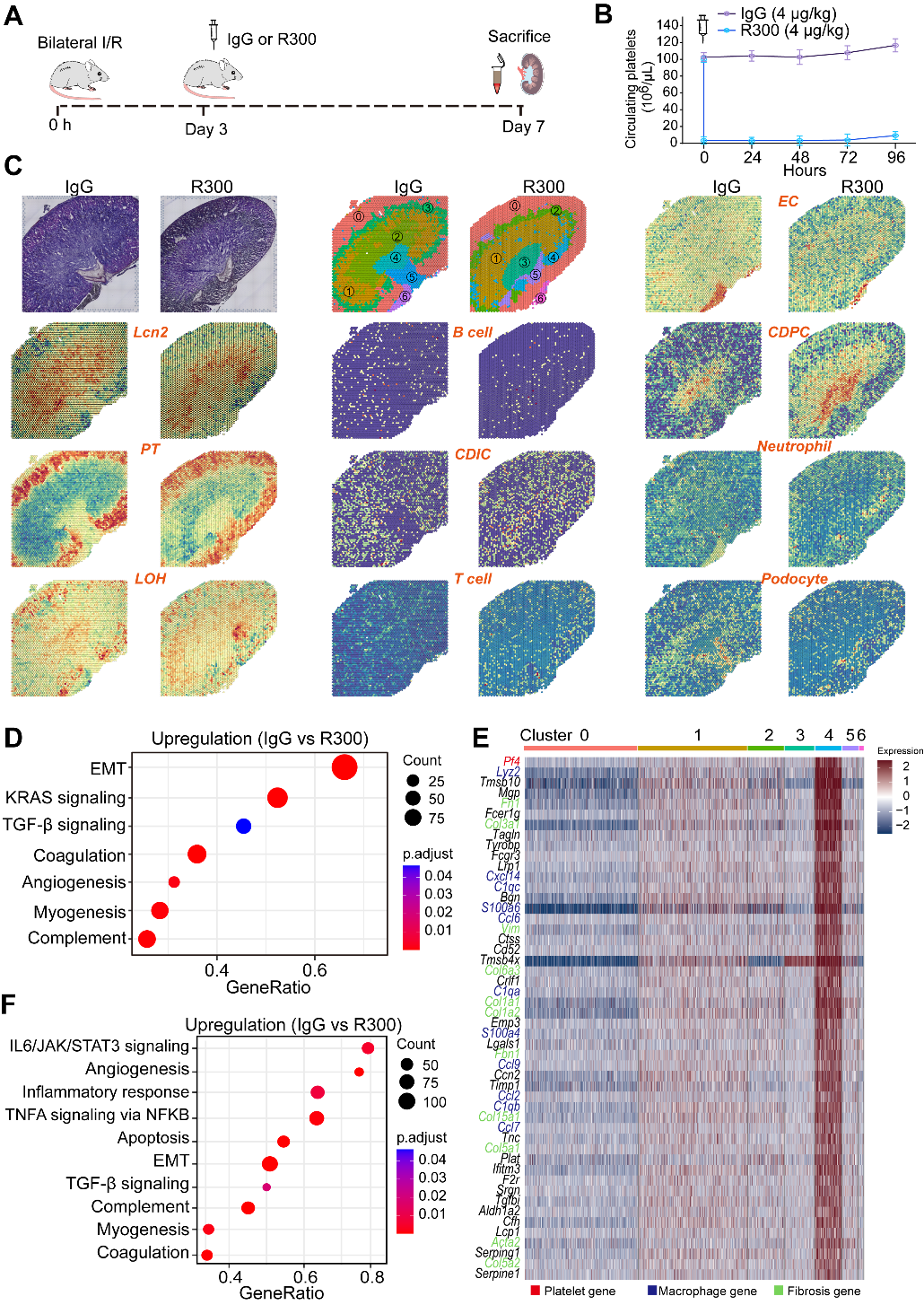


**Figure S2.** Platelet depletion accelerates kidney injury resolution. A) Schematic of the experimental timeline for the establishment of kidney biliteral I/R injury and the following drug treatment. B) C57BL/6J mice were intravenously injected with 4 μg/g R300 or the same amount of isotype IgG. The circulating platelet counts were determined at 0, 24, 48, 72, and 96 hours (*n* = 5 per group). C) Hematoxylin and eosin (H&E) staining of kidney sections and unbiased clustering of the spatial transcriptome (ST) spots of IgG or R300-treated I/R kidneys. Spatial feature plots showing the spatial localization of each cell type in ST spots of IgG or R300-treated I/R kidneys by using its signature genes. D) GESA enrichment analyses were performed on differentially expressed genes by using spatial transcriptomic data in Figure 2I. The dot plot shows the upregulated GSEA hallmark terms in IgG as compared to R300 treated I/R kidneys. E) The spatial expression matrix in cluster 4 of the R300-treated kidney was extracted, and the differentially expressed genes in cluster 4 were analyzed as compared to the other clusters (0, 1, 2, 3, 5,6). Heatmap showing the expression of the top 50 genes upregulated in cluster 4 in R300 treated kidney. Platelet, macrophage, and fibrosis-related genes are highlighted in red, blue, and green font, respectively.


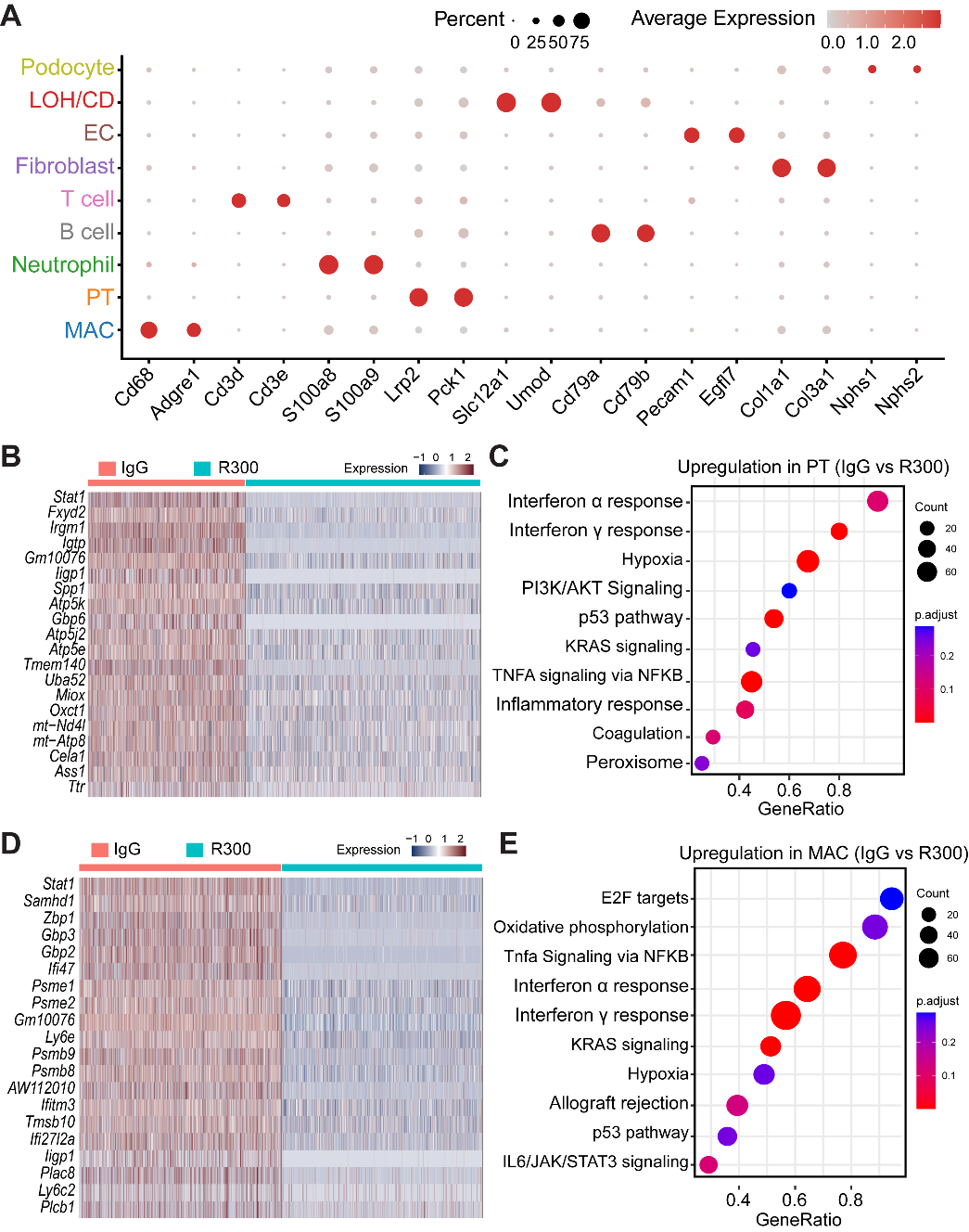


**Figure S3.** Platelet THBS signaling is a potential linker between macrophage and fibroblast for kidney fibrosis progression. A) The dot plot shows expressions of two representative gene markers for each cell type based on the scRNA data in Figure 3A. B) Single-cell mRNA expressions of PT between IgG and R300-treated I/R kidneys were compared. Heatmap showing the top 20 genes upregulated in PT of the IgG-treated kidneys as compared to the R300-treated I/R kidneys. C) The differentially expressed genes in (B) were used to perform GSEA enrichment analysis. The dot plot shows the upregulated GSEA hallmark terms in PT. D) Single-cell mRNA expressions of macrophage between IgG and R300-treated I/R kidneys were compared. Heatmap showing the top 20 genes upregulated in the macrophage of the IgG-treated kidneys compared to the R300-treated I/R kidneys. E) The differentially expressed genes in (D) were used to perform GSEA enrichment analysis. The dot plot shows the upregulated GSEA hallmark terms in macrophage.


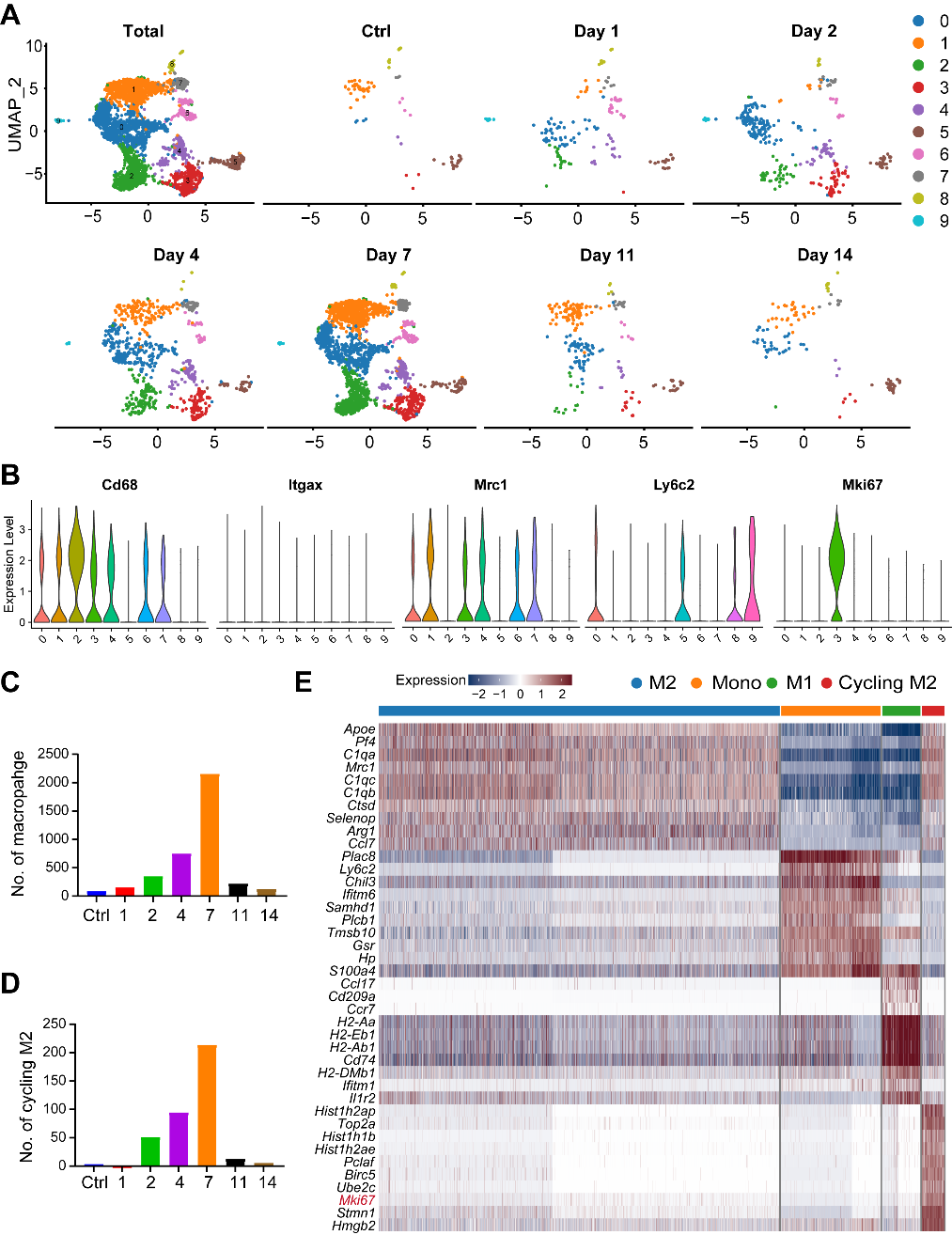


**Figure S4.** Identification of cycling M2-like macrophage with profibrotic feature. A) Wild-type C57BL/6J mice were subjected to kidney I/R surgery. At different times after the surgery, mice were sacrificed and kidney tissues were harvested for single-cell-RNA-sequencing experiments. UMAP projection of all mouse macrophages of kidneys from different groups as indicated. B) violin plot shows the expressions of gene markers in different clusters. C, D) quantification of the numbers of macrophages (C) and cycling macrophages (D) in different groups. E) The heatmap shows each cell type's top 10 exclusively expressed genes.


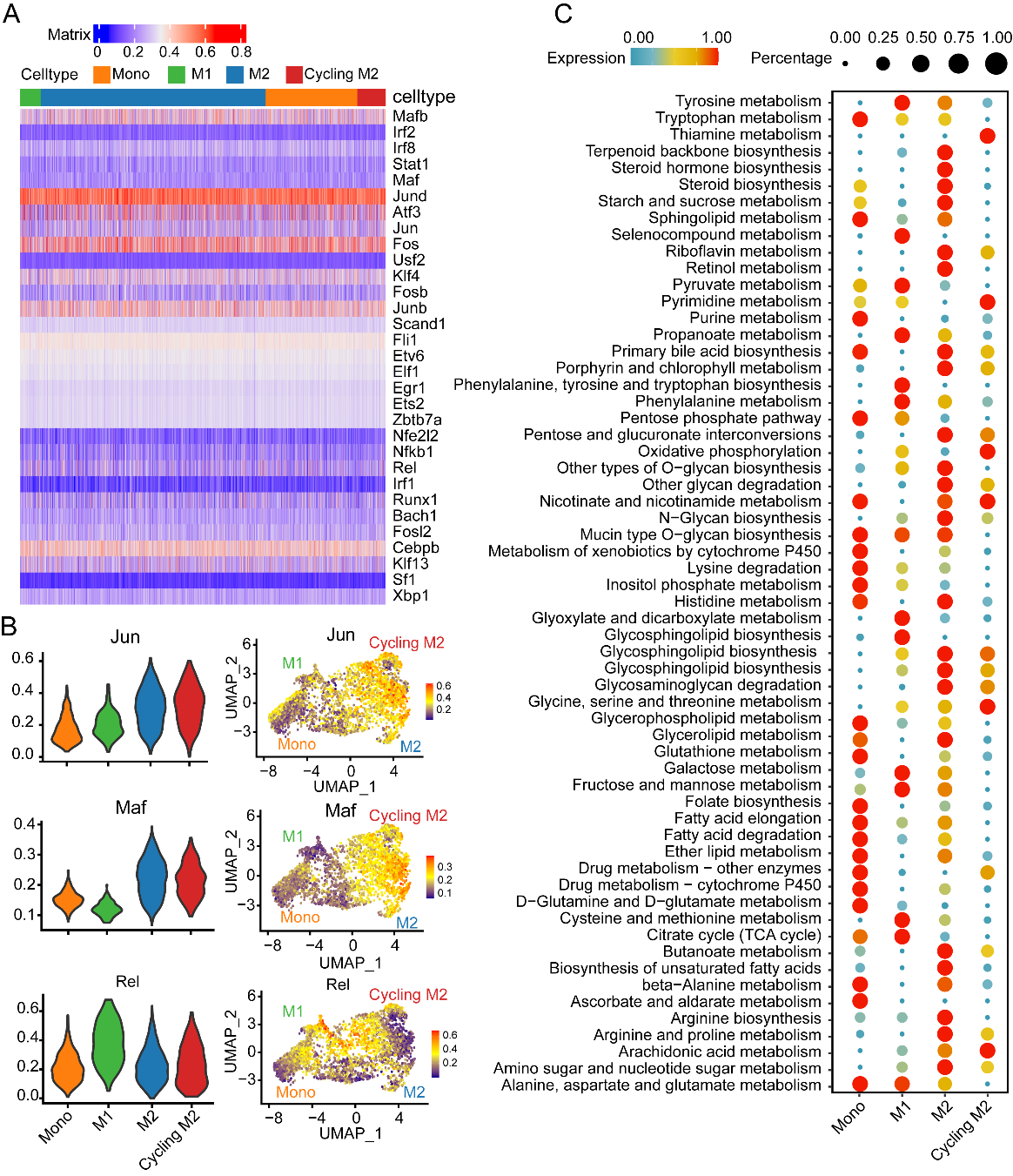


**Figure S5.** Platelet provokes cycling M2 macrophages. A) The heatmap shows the transcriptional factors (TF) in the four cell types analyzed by the R package “SCENIC”. B) The feature plot and violin show the expressions of transcriptional factors Jun, Maf, and Rel in the four cell types. C) The dot plot shows the comparison of metabolic pathways in the four cell types analyzed by the R package “scMetabolism”.


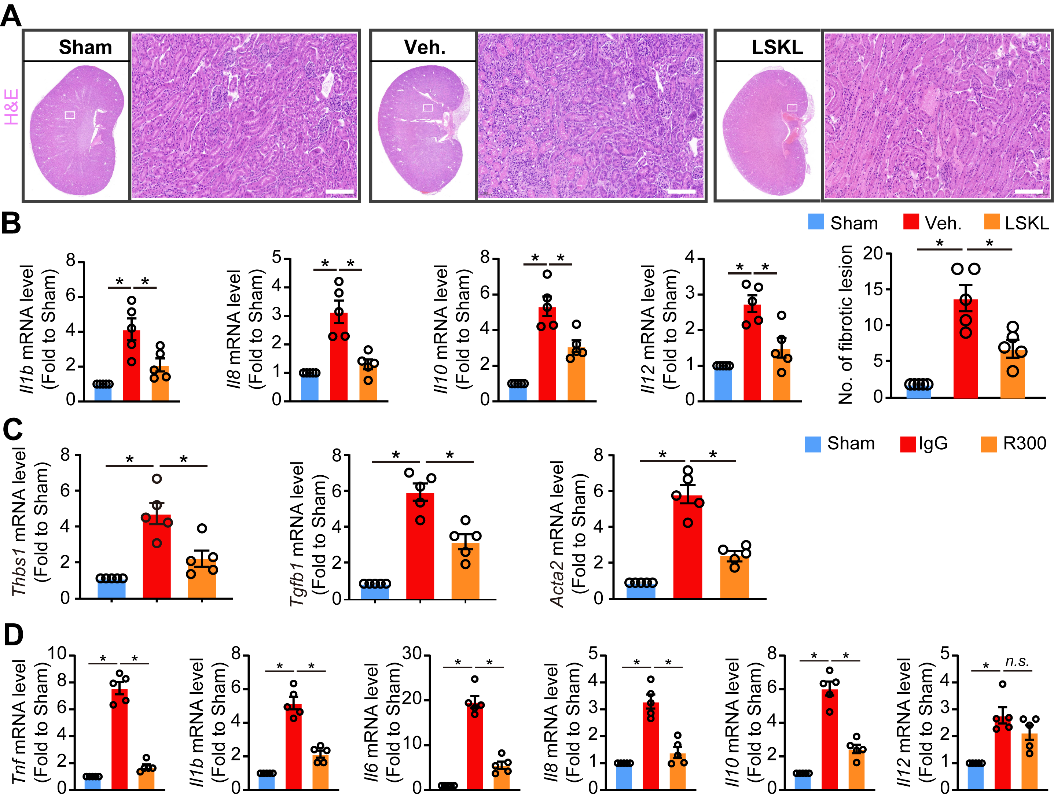


**Figure S6.** A peptide antagonist of platelet THBS1 reduces cycling M2 and ameliorates fibrosis. Mice received the kidney I/R injury with or without THBS-1 inhibitor LSKL treatment. 21 days after the I/R injury, mice were sacrificed and kidney tissues were harvested for qRT-PCR analysis and histopathology. A) Representative the whole kidney picture and staining images of H&E from each group were displayed, Scale bar = 50 μm. B) The relative mRNA expression of *Il1b*, *Il8, Il10* and *Il12* were analyzed. C, D) The relative mRNA expression of *Thbs1, Tgfb1, Acta2, Tnf, Il6, Il1b, Il8, Il10,* and *Il12* were analyzed in the kidneys of the indicated three groups (*n* = 5 mice per group). The statistics were analyzed using an unpaired two-tailed Student’s t-test, **p* < 0.05.


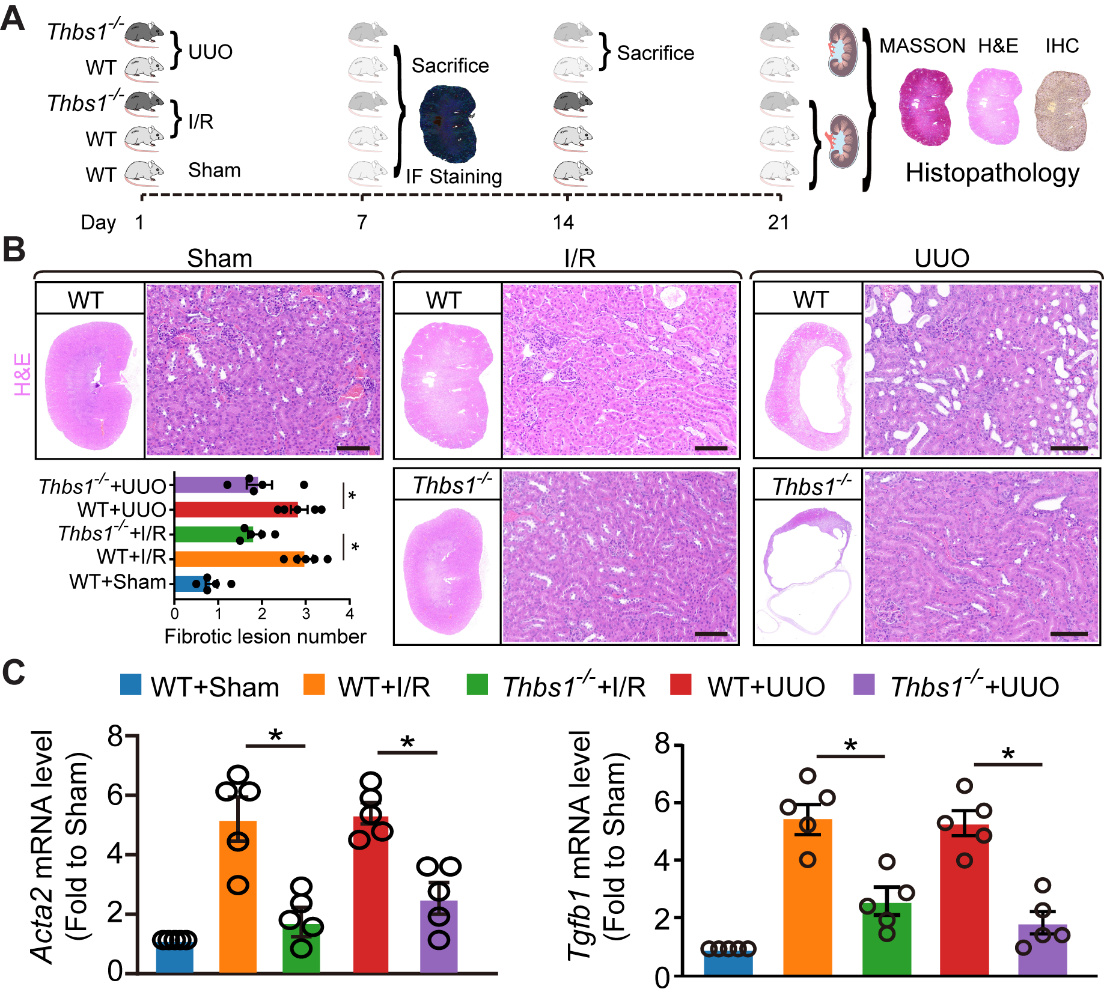


**Figure S7.** Genetic deletion of *Thbs1* attenuates kidney fibrosis. A) Schematic of the timeline for the establishment of kidney biliteral I/R injury or UUO model and the following histopathological experiments including H&E, IHC, MASSON, and IF staining. B) Wild-type or *Thbs1* knockout mice were subjected to the surgery for bilateral I/R kidney injury or UUO and housed for 21 days and 14 days respectively. After that, mice were sacrificed and kidney tissues were harvested for H&E staining. Representative images and the quantifications of H&E staining in the kidney, Scale bar: 50 μm. C) The relative mRNA expression of *Tgfb1* and *Acta2* were analyzed in the kidneys of the indicated groups (*n* = 5 mice per group).

**Table S1**. The clinical information of 102 patients recruited in this study.

| **Case No.** | **Gender^1^** | **Age (Months)** | **Disease^2^** | **Weight (kg)** | **SCr (μmol/L)** | **SCr (H, L)** | **PLT (10*9/L)** | **PLT (H, L)** | **AKI (Y, N)** |
| --- | --- | --- | --- | --- | --- | --- | --- | --- | --- |
| Case 1 | M | 7 | VSD | 8.2 | 22.00 | L | 166.00 | L | N |
| Case 2 | M | 9 | ASD | 8 | 18.00 | L | 202.00 | L | N |
| Case 3 | M | 6 | ToF | 6.1 | 27.00 | L | 203.00 | L | N |
| Case 4 | M | 3 | VSD | 5.5 | 13.00 | L | 209.00 | L | N |
| Case 5 | F | 22 | ASD | 11.5 | 25.00 | L | 213.00 | L | N |
| Case 6 | M | 8 | TGA | 6.9 | 23.00 | L | 215.00 | L | N |
| Case 7 | M | 22 | ASD | 20 | 29.00 | L | 226.00 | L | N |
| Case 8 | M | 25 | ASD | 10 | 26.00 | L | 232.00 | L | N |
| Case 9 | F | 11 | TGA | 5.1 | 27.00 | L | 249.00 | L | N |
| Case 10 | M | 21 | VSD | 11.5 | 19.00 | L | 251.00 | L | N |
| Case 11 | F | 3 | VSD | 5.2 | 17.00 | L | 251.00 | L | N |
| Case 12 | M | 19 | VSD | 12 | 26.00 | L | 254.00 | L | N |
| Case 13 | F | 14 | ASD | 10 | 25.00 | L | 254.00 | L | N |
| Case 14 | M | 6 | VSD | 6.9 | 24.00 | L | 260.00 | L | N |
| Case 15 | F | 13 | ASD | 10.5 | 22.00 | L | 260.00 | L | N |
| Case 16 | M | 0.2 | CoA | 3.3 | 21.00 | L | 263.00 | L | N |
| Case 17 | F | 7 | VSD | 7 | 25.00 | L | 266.00 | L | N |
| Case 18 | F | 3 | VSD | 4.4 | 26.00 | L | 270.00 | L | N |
| Case 19 | M | 25 | ASD | 11 | 26.00 | L | 270.00 | L | N |
| Case 20 | F | 8 | VSD | 6.5 | 22.00 | L | 276.00 | L | N |
| Case 21 | F | 5 | VSD | 5.1 | 26.00 | L | 279.00 | L | N |
| Case 22 | F | 5 | VSD | 5.5 | 17.00 | L | 279.00 | L | N |
| Case 23 | M | 25 | VSD | 14 | 27.00 | L | 281.00 | L | N |
| Case 24 | F | 2 | VSD | 4.3 | 27.00 | L | 284.00 | L | N |
| Case 25 | M | 30 | VSD | 16 | 27.00 | L | 285.00 | L | N |
| Case 26 | F | 1 | VSD | 3.9 | 29.00 | L | 291.00 | L | N |
| Case 27 | M | 12 | VSD | 9.5 | 24.00 | L | 296.00 | L | N |
| Case 28 | M | 5 | VSD | 5.5 | 24.00 | L | 301.00 | L | N |
| Case 29 | F | 21 | VSD | 11 | 21.00 | L | 301.00 | L | N |
| Case 30 | F | 4 | VSD | 4.9 | 23.00 | L | 302.00 | L | N |
| Case 31 | M | 5 | ToF | 6.5 | 24.00 | L | 311.00 | L | N |
| Case 32 | F | 4 | VSD | 6 | 22.00 | L | 311.00 | L | N |
| Case 33 | M | 16 | VSD | 11.5 | 23.00 | L | 315.00 | L | N |
| Case 34 | M | 28 | VSD | 12 | 27.00 | L | 317.00 | L | N |
| Case 35 | M | 3 | VSD | 6 | 24.00 | L | 318.00 | L | N |
| Case 36 | F | 3 | VSD | 14 | 23.00 | L | 321.00 | L | N |
| Case 37 | F | 4 | VSD | 6.5 | 23.00 | L | 323.00 | L | N |
| Case 38 | F | 24 | VSD | 14 | 28.00 | L | 327.00 | L | N |
| Case 39 | F | 24 | VSD | 12 | 24.00 | L | 328.00 | L | N |
| Case 40 | M | 2 | VSD | 5 | 20.00 | L | 330.00 | L | N |
| Case 41 | M | 21 | VSD | 11 | 26.00 | L | 337.00 | L | N |
| Case 42 | F | 13 | CoA | 8.2 | 20.00 | L | 339.00 | L | N |
| Case 43 | F | 26 | VSD | 11 | 23.00 | L | 344.00 | L | N |
| Case 44 | F | 2 | VSD | 4 | 24.00 | L | 345.00 | L | N |
| Case 45 | F | 1 | CoA | 4 | 31.00 | H | 250.00 | L | N |
| Case 46 | F | 7 | VSD | 8.9 | 31.00 | H | 266.00 | L | N |
| Case 47 | M | 7 | ToF | 8 | 32.00 | H | 268.00 | L | N |
| Case 48 | M | 21 | TGA | 12.5 | 31.00 | H | 296.00 | L | N |
| Case 49 | F | 32 | ASD | 11.5 | 30.00 | H | 303.00 | L | N |
| Case 50 | M | 1 | CoA | 5 | 32.00 | H | 343.00 | L | N |
| Case 51 | M | 3 | VSD | 6 | 19.00 | L | 353.00 | H | N |
| Case 52 | F | 1 | VSD | 3.2 | 13.00 | L | 358.00 | H | N |
| Case 53 | M | 9 | TGA | 6.8 | 29.00 | L | 363.00 | H | N |
| Case 54 | M | 17 | ASD | 20 | 22.00 | L | 380.00 | H | N |
| Case 55 | F | 3 | VSD | 4.7 | 21.00 | L | 381.00 | H | N |
| Case 56 | F | 3 | VSD | 4.8 | 25.00 | L | 393.00 | H | N |
| Case 57 | F | 3 | ToF | 4.4 | 27.00 | L | 410.00 | H | N |
| Case 58 | M | 6 | ToF | 9.5 | 28.00 | L | 439.00 | H | N |
| Case 59 | F | 11 | ASD | 7.4 | 31.00 | H | 365.00 | H | N |
| Case 60 | F | 2 | VSD | 4 | 32.00 | H | 368.00 | H | N |
| Case 61 | F | 12 | ASD | 10 | 32.00 | H | 368.00 | H | N |
| Case 62 | M | 10 | VSD | 11 | 31.00 | H | 411.00 | H | N |
| Case 63 | F | 2 | VSD | 3.4 | 32.00 | H | 454.00 | H | N |
| Case 64 | M | 25 | ASD | 15 | 29.00 | L | 235.00 | L | Y |
| Case 65 | M | 4 | VSD | 6.9 | 27.00 | L | 313.00 | L | Y |
| Case 66 | M | 0 | CoA | 3.6 | 35.00 | H | 153.00 | L | Y |
| Case 67 | F | 0.3 | TGA | 3.5 | 44.00 | H | 229.00 | L | Y |
| Case 68 | M | 23 | ASD | 12.5 | 41.00 | H | 230.00 | L | Y |
| Case 69 | F | 2 | VSD | 4 | 35.00 | H | 233.00 | L | Y |
| Case 70 | F | 3 | VSD | 4.4 | 33.00 | H | 255.00 | L | Y |
| Case 71 | M | 10 | ToF | 6.5 | 48.00 | H | 274.00 | L | Y |
| Case 72 | M | 1 | VSD | 3.9 | 35.00 | H | 276.00 | L | Y |
| Case 73 | F | 1 | VSD | 3.8 | 44.00 | H | 278.00 | L | Y |
| Case 74 | M | 7 | VSD | 6.4 | 49.00 | H | 282.00 | L | Y |
| Case 75 | F | 4 | VSD | 5.5 | 34.00 | H | 286.00 | L | Y |
| Case 76 | M | 3 | ToF | 6.4 | 34.00 | H | 300.00 | L | Y |
| Case 77 | M | 31 | VSD | 11 | 34.00 | H | 306.00 | L | Y |
| Case 78 | F | 2 | VSD | 4 | 33.00 | H | 309.00 | L | Y |
| Case 79 | F | 20 | VSD | 12 | 49.00 | H | 324.00 | L | Y |
| Case 80 | F | 29 | VSD | 11.3 | 34.00 | H | 324.00 | L | Y |
| Case 81 | M | 27 | ASD | 14 | 31.00 | H | 326.00 | L | Y |
| Case 82 | F | 1 | VSD | 3.7 | 52.90 | H | 336.00 | L | Y |
| Case 83 | F | 15 | TGA | 8.5 | 43.00 | H | 339.00 | L | Y |
| Case 84 | M | 2 | VSD | 6 | 28.00 | L | 368.00 | H | Y |
| Case 85 | M | 31 | VSD | 12 | 41.00 | H | 350.00 | H | Y |
| Case 86 | M | 3 | VSD | 5 | 74.00 | H | 360.00 | H | Y |
| Case 87 | F | 26 | VSD | 5.2 | 63.70 | H | 360.00 | H | Y |
| Case 88 | F | 2 | VSD | 4.3 | 33.00 | H | 365.00 | H | Y |
| Case 89 | M | 0.5 | TGA | 3.2 | 41.00 | H | 373.00 | H | Y |
| Case 90 | F | 6 | ASD | 7.6 | 37.00 | H | 373.00 | H | Y |
| Case 91 | F | 3 | VSD | 5.4 | 36.00 | H | 377.00 | H | Y |
| Case 92 | M | 1 | CoA | 3.2 | 34.00 | H | 380.00 | H | Y |
| Case 93 | M | 3 | VSD | 5.5 | 34.00 | H | 381.00 | H | Y |
| Case 94 | M | 14 | ASD | 9.8 | 30.00 | H | 388.00 | H | Y |
| Case 95 | F | 7 | ToF | 6.5 | 30.00 | H | 418.00 | H | Y |
| Case 96 | M | 5 | VSD | 6 | 34.00 | H | 421.00 | H | Y |
| Case 97 | F | 3 | VSD | 4 | 42.00 | H | 427.00 | H | Y |
| Case 98 | F | 3 | ToF | 5.3 | 34.00 | H | 436.00 | H | Y |
| Case 99 | F | 17 | VSD | 9 | 30.00 | H | 436.00 | H | Y |
| Case 100 | F | 2 | VSD | 4.5 | 42.00 | H | 454.00 | H | Y |
| Case 101 | F | 12 | VSD | 8 | 33.00 | H | 507.00 | H | Y |
| Case 102 | M | 3 | TGA | 3.8 | 62.00 | H | 518.00 | H | Y |

^1^ M: male, F: female;

^2^ VSD: Ventricular Septal Defect, ToF: Tetralogy of Fallot, ASD: Atrial Septal Defect, CoA: Coarctation of Aorta, TGA: Transposition of the Great Arteries;

**Table S2**. A detailed demographic information of the enrolled 102 patients.

| \| **Characteristic** \| **Total** \| **AKI** \| **Non-AKI** \| ***p*-value**^1^ \| \| --- \| --- \| --- \| --- \| --- \| \| **Number of patients** \| *n* = 102 \| *n* = 39 \| *n* = 63 \|  \| \| **Age (months, meanSEM)**  \| 9.99 ± 0.93 \| 9.00 ± 1.60 \| 10.06 ± 1.13 \| 0.403 \| \| **Weight (kg, meanSEM)** \| 7.68 ± 0.37 \| 6.70 ± 0.52 \| 8.29 ± 0.49 \| 0.035 \| \| **Gender** \|  \|  \|  \| 0.538 \| \| Male \| 49 \| 19 \| 30 \|  \| \| Female \| 53 \| 20 \| 33 \|  \| \| **Disease**^2^ \|  \|  \|  \| 0.909 \| \| VSD \| 63 \| 24 \| 39 \|  \| \| ToF \| 9 \| 4 \| 5 \|  \| \| ASD \| 16 \| 5 \| 11 \|  \| \| CoA \| 6 \| 2 \| 4 \|  \| \| TGA \| 8 \| 4 \| 4 \|  \| |
| --- | --- | --- | --- | --- | --- | --- | --- | --- | --- | --- | --- | --- | --- | --- | --- | --- | --- | --- | --- | --- | --- | --- | --- | --- | --- | --- | --- | --- | --- | --- | --- | --- | --- | --- | --- | --- | --- | --- | --- | --- | --- | --- | --- | --- | --- | --- | --- | --- | --- | --- | --- | --- | --- | --- | --- | --- | --- | --- | --- | --- | --- | --- | --- | --- | --- |

^1^ Fisher Exact Probability Test

^2^ VSD: Ventricular Septal Defect, ToF: Tetralogy of Fallot, ASD: Atrial Septal Defect, CoA: Coarctation of Aorta, TGA: Transposition of the Great Arteries;

**Table S3**. Primers were used for quantitative real-time PCR in this study.

| **Gene** | **Forward (5’ to 3’)** | **Reverse (5’ to 3’)** |
| --- | --- | --- |
| Human _*Il1b* | ACTCACCTCTTCAGAACGAATTG | CCATCTTTGGAAGGTTCAGGTTG |
| Mouse_*Il8* | GCTACTGTTGGCCCAATTACTAAC | TGTTCTCAGGTCTCCCAAATGAA |
| Mouse_*Il12* | GACCAAACCAGCACATTGAAGAC | TGATTCTGAAGTGCTGCGTTGAT |
| Mouse_*Thbs1* | CATGTCGACATGGAGCTCCTGCGGGGACTAGGTGTC | GAGAAGCTTTAGGAATCTCGACACTCGTATTTCATGTC |
| Mouse_*Tgfb1* | GCGGACTACTATGCTAAAGAGGT | GCTTCCCGAATGTCTGACGTATT |
| Mouse_*Acta2* | CCAGCTATGTGTGAAGAGGAAGA | TTGGTGATGATGCCGTGTTCTAT |
| Mouse_*Il6* | TCTGGGAAATCGTGGAAATGAG | TCTCTGAAGGACTCTGGCTTTGTC |
| Mouse_*Tnf* | GACCCTCACACTCAGATCATCTT | CCTTGAAGAGAACCTGGGAGTAG |
| Mouse_*Gapdh* | AGGTCGGTGTGAACGGATTTG | GGGGTCGTTGATGGCAACA |

**Table S4**. Reagents and antibodies were used in this study.

| **Antibody** | **Source** | **Identifier** | **Dilution** | **Validation** |
| --- | --- | --- | --- | --- |
| α-SMA | Abcam | ab5694 | 1:200 for IHC | https://www.abcam.com/products/primary-antibodies/alpha-smooth-muscle-actin-antibody-ab5694.html |
| THBS1 | Abcam | ab267388 | 1:200 for IF | https://www.abcam.com/products/primary-antibodies/thrombospondin-1-antibody-epr22927-54-ab267388.html |
| THBS1 | Cell signaling | 37879 | 1:500 for WB | https://www.cellsignal.com/products/primary-antibodies/thrombospondin-1-d7e5f-rabbit-mab/37879 |
| R300 | Emfret Analytics | R300 | No applicable | https://www.emfret.com/fileadmin/user_upload/Datasheets/R300.pdf |
| β-actin | Cell signaling | 4790 | 1:1000 for WB | https://www.cellsignal.com/products/primary-antibodies/b-actin-13e5-rabbit-mab/4970 |
| CD206 | Abcam | ab64693 | 1:200 for IF | https://www.abcam.com/products/primary-antibodies/mannose-receptor-antibody-ab64693.html |
| Ki67 | Abcam | ab16667 | 1:200 for IF | https://www.abcam.com/products/primary-antibodies/ki67-antibody-sp6-ab16667.html |
